# Metabolic Memory in Cardiovascular Disease: Encoding, Propagation, and Therapeutic Targeting

**DOI:** 10.1002/advs.77260

**Published:** 2026-08-27

**Authors:** Cheng Cheng, Minghui Tang, Zhaobo Zhang, Ruoyi Qu, Jianfei Pei, Tian Liu, Siyao Qu, Shilong You, Shenshen Cui, Wenke Wang, Rui Zhao, Yingxian Sun, Naijin Zhang

**Affiliations:** ^1^ Department of Central Laboratory The First Hospital of China Medical University Shenyang Liaoning China; ^2^ Department of Cardiology The First Hospital of China Medical University Shenyang Liaoning China; ^3^ Department of Internal Medicine The First Hospital of China Medical University Shenyang Liaoning China; ^4^ Institute of Health Professions Education Assessment and Reform China Medical University Shenyang Liaoning China; ^5^ Department of Medical Genetics China Medical University Shenyang Liaoning China; ^6^ Center of Reproductive Medicine Shengjing Hospital of China Medical University Shenyang Liaoning China; ^7^ Department of Forensic Pathology School of Forensic Medicine China Medical University Shenyang Liaoning China

**Keywords:** atherosclerosis, diabetic cardiomyopathy, epigenetic regulation, heart failure, metabolic memory, trained immunity

## Abstract

Cardiovascular risk in metabolic disease persists long after the initiating metabolic abnormalities are corrected, a phenomenon termed metabolic memory. The DCCT/EDIC cohort is illustrative: early glycemic control produced cardiovascular protection that peaked within a decade and left a lasting legacy. The same strategy applied after prolonged hyperglycemia, however, has not reproduced this benefit. This conceptual Review proposes a framework in which such persistent risk arises from four distinct processes: encoded epigenetic memory, irreversible structural damage, chronic input from dysfunctional organs, and delayed tissue remodeling, each with a different therapeutic logic. Persistence of these encoded marks is established most directly in immune‐lineage cells; its extension to cardiomyocytes remains a working hypothesis. Only encoded memory is accessible to chromatin‐directed reversal, and only before metabolic stress exhausts the erasure machinery that keeps marks revisable, a time‐dependence proposed to explain why early intervention succeeds where late intervention fails. Clinical efficacy therefore may depend on engaging the substrate maintaining pathology rather than normalizing a surrogate biomarker, a substrate‐alignment principle consistent with the divergent outcomes of recent cardiometabolic trials. We apply the framework to atherosclerosis, heart failure, and diabetic cardiomyopathy, grade its claims by a three‐tier evidence classification, and specify testable predictions that could refute it.

## Graphical abstract

Cardiovascular risk often persists after metabolic abnormalities are corrected. This conceptual Review frames such persistence as metabolic memory, encoded through a narrowing therapeutic window from reversible marks to irreversible damage, with continuous input from peripheral organs. It proposes that lasting benefit may depend on engaging a causal substrate before encoding becomes irreversible, offering a logic for stage‐matched cardiovascular therapy.

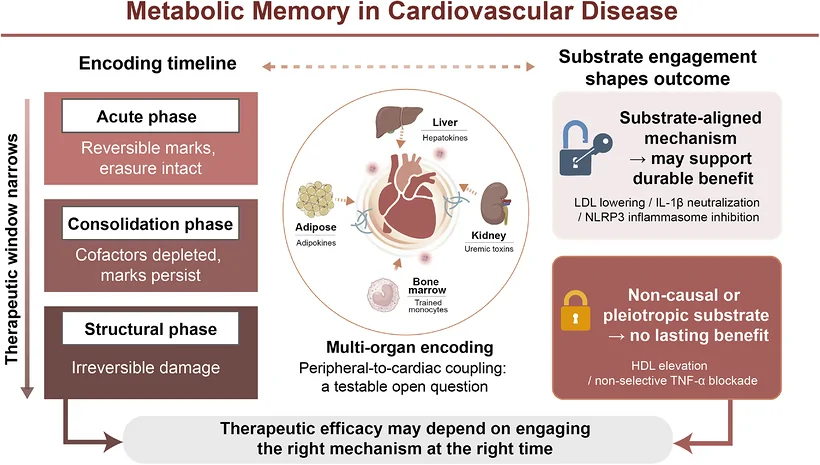

## 1 Introduction

The Diabetes Control and Complications Trial (DCCT) and its Epidemiology of Diabetes Interventions and Complications (EDIC) follow‐up demonstrated that a mean of 6.5 years of intensive glycemic control in type 1 diabetes conferred cardiovascular protection that peaked within the first decade after glucose convergence between treatment groups and left a smaller, lasting legacy [1, 2]. In contrast, intensive glucose lowering initiated after prolonged hyperglycemic exposure in type 2 diabetes yielded no significant cardiovascular benefit despite achieving guideline targets, as demonstrated in the VADT and ACCORD trials [3, 4]. In ACCORD, intensive glycemic control was additionally associated with increased all‐cause mortality, leading to early termination of that treatment arm [4]. These observations define a central paradox: early metabolic intervention produces lasting cardiovascular benefit, yet the same intervention fails when applied after prolonged metabolic dysregulation.

Both halves of this paradox point to the same underlying property: once metabolic stress has been encoded, its cardiovascular consequences persist after the initiating exposure is corrected. Benefit endures when the exposure is brief, and risk endures when it is prolonged. This persistence of glucose‐driven risk after glycemic correction, first demonstrated in the DCCT/EDIC cohort [1, 2], is the original instance of what the diabetes literature has termed glycemic memory [5, 6]. The same persistence, however, is not confined to glucose. We use metabolic memory as an umbrella term: molecular programs, encoded through epigenetic modification and immune‐cell reprogramming, that persist after the initiating perturbation has resolved, whether driven by hyperglycemia, dyslipidemia, or trained immunity. A single category is retained because these triggers converge on the same writing‐and‐erasure machinery, generating persistence through a single class of mechanism rather than several distinct ones. Where evidence is specific to glucose, we use the narrower term glycemic memory.

Encoded memory of this kind, however, is only one of four mechanistically distinct processes that sustain cardiovascular pathology after metabolic normalization, and it is the only one that stores reversible information. The other three do not. Irreversible structural changes such as advanced glycation end product (AGE) crosslinks and vascular calcification represent largely irreversible matrix alterations rather than stored information. Sustained pathological input from dysfunctional organs reflects ongoing exposure rather than autonomous encoding. Delayed tissue remodeling reflects repair kinetics rather than persistent programming. These categories frequently coexist in individual patients, and distinguishing among them carries direct therapeutic consequences: encoded memory is the only category accessible to reversal through chromatin‐directed intervention.

Transient hyperglycemia produces persistent histone modifications in vascular endothelial cells [7], with analogous persistence of DNA methylation changes at loci enriched for myeloid regulatory elements in the DCCT/EDIC cohort [8]. Trained immunity imprints durable chromatin marks in myeloid progenitors that resist metabolic normalization [9]. What remains less developed is how these mechanisms are organized for therapeutic decision‐making. Prior reviews have addressed the epigenetic basis of metabolic memory [10, 11], its intersection with trained immunity [9], and its mechanistic basis in diabetic vascular complications specifically [12]. Dong et al. [10] survey metabolic memory broadly across metabolic disease, and Chen et al. [11] review the epigenetics of diabetic complications with a predominantly renal emphasis. Neither organizes these mechanisms by therapeutic substrate nor maps how reversibility narrows across disease stages, the two axes on which therapeutic decisions depend. Beyond this organizational gap, a substantive question remains unresolved: whether peripheral organ mediator exposure directly drives cardiac chromatin remodeling, or whether cardiac modifications arise independently.

Building on this prior work, this Review makes three contributions. First, it classifies persistent cardiovascular risk by therapeutic substrate rather than by molecular mechanism, separating encoded memory, which is reversible, from structural, input‐driven, and remodeling processes, which are not. Second, it maps a temporal hierarchy of encoding onto the therapeutic window proposed to be available at each disease stage. Third, it distinguishes two persistence mechanisms with divergent therapeutic implications: immune‐lineage memory, regenerated continuously through progenitor reprogramming in bone marrow, and cardiomyocyte memory, persisting passively through cell longevity. We trace how metabolite signals are converted into stable chromatin states and how encoded programs propagate through intercellular and inter‐organ networks. We then apply the framework to atherosclerosis, heart failure, and diabetic cardiomyopathy, grade mechanistic claims by evidence strength (Box 1), and identify experiments whose outcomes could refute the model.

This Review is narrative in scope. The literature was identified through iterative PubMed searches up to June 2026 and screening of key reference lists, centering on metabolic and glycemic memory, trained immunity, chromatin modifications, cofactor metabolism, inter‐organ signaling, and cardiometabolic trials. Studies were prioritized by directness of evidence and by relevance to the two organizing axes of this Review, therapeutic substrate and strength of evidence, with emphasis on human longitudinal and interventional studies where available and on mechanistic work using direct causal manipulation. Key mechanistic claims were graded by the criteria in Box 1.

## 2 Molecular Encoding of Metabolic Memory: From Metabolite Sensing to Chromatin Consolidation

The central question is how metabolite fluctuations that resolve within hours under physiological conditions become translated, under chronic metabolic stress, into molecular programs persisting for decades. Table 1 systematizes the pathway‐modification relationships discussed below, and Box 1 classifies the corresponding mechanistic claims by evidence strength.

**TABLE 1:** Chromatin modifications in cardiovascular metabolic memory. Chromatin modifications encode cardiovascular metabolic memory through direct metabolite‐chromatin coupling. Entries are organized by metabolic source pathway; metabolites that feed acylation through a shared acetyl‐CoA pool are grouped together as an acylation hub rather than by their separate source pathways. Entries are restricted to metabolite‐derived histone or DNA modifications with demonstrated or mechanistically grounded persistence (memory‐capable); histone modifications that track substrate levels in real time without demonstrated persistence (e.g., succinylation and malonylation), and metabolite‐driven modifications of non‐histone proteins such as signaling and transcriptional regulators (e.g., O‐GlcNAcylation; Section 4.3) are excluded from this table. Two entries warrant explicit qualification. For lactylation, cardiac persistence is inferred by analogy to the demonstrated persistence of this mark in immune‐lineage cells (e.g., trained immunity); for β‐hydroxybutyrylation, inclusion rests on direct cardiac histone‐mark evidence (H3K9bhb at the *CPT1A* locus after myocardial infarction, in cardiac endothelial cells), though stimulus‐independent persistence remains undemonstrated. The temporal ranges for both reflect stimulus‐coupled dynamics rather than demonstrated stimulus‐independent persistence. Histone modifications use the protein‐residue format (e.g., H3K18la denotes histone H3 lysine 18 lactylation). In the writer field, “n/a” denotes erasure‐impaired entries, where the modification accumulates through loss of removal rather than active installation, and “Non‐enzymatic” denotes direct chemical installation without an enzyme. Reversal‐strategy maturity labels (preclinical, hypothetical) indicate the current evidence level for each proposed strategy; no strategy has yet achieved RCT‐validated cardiovascular benefit through demonstrated reversal of an encoded epigenetic memory substrate in human myocardium. The Key Evidence column indicates organism and tissue type for each cited reference.

Metabolic source	Modification	Writer/eraser	Chromatin targets	Significance	Temporal	Reversal strategy (maturity)	Key evidence
**Glycolysis**							
Lactate	Lactylation	p300, GCN5/HDAC1‐3	H3K18la	real‐time lactate sensing; reparative gene activation	concentration‐dependent (real‐time–subacute)	LDHA or MCT1 inhibition (preclinical)	[13](*Mφ*); [14](CM); [15](foundational Kla mark; non‐cardiac)
**Acylation hub (multi‐pathway integration)**							
Acetyl‐CoA (glycolysis, TCA, ketones, acetate)	Acetylation (general)	p300/SIRT1, HDACs	H3K27ac; H3K9ac	metabolic memory; inflammatory gene activation	Hours–weeks/years	NAD^+^ precursors (preclinical)	[16](*Mφ*); [17](foundational ACLY‐acetylation link; non‐cardiac)
Citrate → nuclear ACLY	Acetylation (nuclear)	Nuclear ACLY‐p300/HDACs	H3K27ac (fibrotic loci)	myofibroblast persistence; cardiac remodeling	Hours–weeks	ACLY inhibition (preclinical)	[18](CF)
NAD^+^ depletion	Acetylation (mito)	Non‐enzymatic(acetyl‐CoA‐driven)/SIRT3 (NAD^+^‐limited)	Mitochondrial enzymes	bioenergetic collapse; ATP depletion; heart failure	Weeks–months	NAD^+^ repletion (preclinical)	[19, 20](CM‐murine; CM‐human HFpEF)
**Methylation (one‐carbon writing / α‐KG‐dependent erasure)**							
SAM (one‐carbon)	DNA methylation (locus hypomethylation)	DNMTs/TETs	3′‐UTR CpG (*TXNIP*)	DCCT legacy; glycemic memory	Days–decades	restoration of methylation (hypothetical)	[8](human, DCCT/EDIC); [21](CM‐human LVAD)
SAM (one‐carbon)	Histone methylation (writing)	Set7/KDMs	H3K4me1 (p65/NF‐κB loci)	persistent pro‐inflammatory transcription	Days	Set7 inhibition (preclinical)	[7](EC‐human, high‐glucose)
α‐KG depletion	DNA methylation (impaired erasure)	n/a/TET1‐3 (α‐KG‐dependent, impaired)	Gene‐body CpG (5mC/5hmC); *TGFBR2*	aberrant gene‐body methylation/hydroxymethylation (diabetic heart)	Days–decades	α‐KG restoration (preclinical)	[22](CM‐rat diabetic)
**Fatty acid oxidation**							
Crotonyl‐CoA	Crotonylation	p300/HDACs	H3K18cr, H2BK12cr	cardiac hypertrophy; ischemia–reperfusion injury	Days–weeks	ECHS1 activation; crotonate therapy (preclinical)	[23, 24](CM‐murine)
**Ketone metabolism**							
β‐Hydroxybutyrate	β‐Hydroxybutyrylation	p300/HDAC1‐2, SIRT3	H3K9bhb (*CPT1A* locus)	post‐MI angiogenic reprogramming (H3K9bhb–*CPT1A*)	concentration‐dependent (real‐time–subacute)	ketone‐flux control (preclinical)	[25](EC‐human, murine post‐MI); [26] (foundational Kbhb mark; non‐cardiac)
**Amino acid metabolism**							
Propionyl‐CoA	Propionylation	p300, CBP, KAT6A/HDACs	H3K23pr, H3K9pr, H3K122pr	fibrosis; diastolic dysfunction	Days‐weeks	BCAA restriction; β‐alanine supplementation (preclinical)	[27](foundational Kpr mark; non‐cardiac); [28, 29](CM‐murine)

**Abbreviations**: ACLY, ATP citrate lyase; BCAA, branched‐chain amino acid; CBP, CREB‐binding protein; CPT1A, carnitine palmitoyltransferase 1A; DCCT, Diabetes Control and Complications Trial; DNMT, DNA methyltransferase; ECHS1, enoyl‐CoA hydratase short chain 1; EDIC, Epidemiology of Diabetes Interventions and Complications; GCN5, general control nonderepressible 5; HDAC, histone deacetylase; HFpEF, heart failure with preserved ejection fraction; KAT6A, lysine acetyltransferase 6A; KDM, lysine demethylase; α‐KG, α‐ketoglutarate; LDHA, lactate dehydrogenase A; MCT, monocarboxylate transporter; MI, myocardial infarction;  NF‐κB, nuclear factor κB; SAM, S‐adenosylmethionine; Set7, SET domain containing lysine methyltransferase 7; SIRT, sirtuin; TCA, tricarboxylic acid; TET, ten‐eleven translocation; TGFBR2, transforming growth factor beta receptor 2; TXNIP, thioredoxin‐interacting protein; 5hmC, 5‐hydroxymethylcytosine; 5mC, 5‐methylcytosine. **Cell type**: CM, cardiomyocytes; CF, cardiac fibroblasts; EC, endothelial cells; Mφ, macrophages/monocytes.

**Box 1: Evidence classification for the metabolic memory framework**

The metabolic memory framework integrates findings from human longitudinal cohorts, animal genetic models, and in vitro mechanistic studies. Because these evidence streams differ in directness, we classify key mechanistic claims into three tiers by the strength of their support.

Tier A includes human longitudinal or interventional studies, animal experiments employing direct causal interventions such as genetic knockout, transplantation, or surgical manipulation, and combinations of animal causal evidence with concordant human observational validation.

Tier B includes findings from in vitro systems, observational human or animal time‐course studies without paired causal manipulation, and claims resting on several mutually consistent lines of evidence, none of which individually meets the Tier A causal criterion.

Tier C includes mechanistic claims consistent with available evidence but not yet directly tested; these require prospective validation before clinical translation.

**Tier A** Demonstrated in human studies or supported by direct causal evidence.

**Table:** 

**Mechanism/observation**	**Evidence type**	**Key references**
Monocyte H3K9ac differences between DCCT conventional‐arm progressors and intensive‐arm non‐progressors persisted at EDIC year 16‐17 and tracked mean DCCT HbA1c.	Human longitudinal cohort (DCCT/EDIC)	Miao et al. [30]
Skin collagen AGE levels at DCCT closeout predicted microvascular complications and were independently associated with subclinical cardiovascular measures across years 1–16 of EDIC follow‐up despite glycemic convergence.	Human longitudinal cohort (DCCT/EDIC)	Genuth et al. [31]; Monnier et al. [32]
Thioredoxin‐interacting protein (TXNIP) 3′‐UTR hypomethylation acquired during DCCT persists into EDIC and statistically mediates the association between prior HbA1c and subsequent complications.	Human longitudinal cohort (DCCT/EDIC)	Chen et al. [8]
Three months of statin therapy fails to reverse monocyte trained immunity or associated epigenetic marks.	Human interventional study	Bekkering et al. [33]
Bone marrow from hyperglycemia‐exposed donors transferred into normoglycemic recipients accelerates atherosclerosis through trained myeloid progenitors.	Animal, causal (bone marrow transplantation)	Edgar et al. [34]
Bone marrow transplantation from heart failure mice causes spontaneous cardiac dysfunction in healthy recipients.	Animal, causal (transplantation)	Nakayama et al. [35]
Global selenoprotein P (SeP) knockout attenuates myocardial ischemia‐reperfusion injury; circulating SeP predicts adverse human heart failure outcomes.	Animal, causal (global knockout; human observational)	Chadani et al. [36]; Takeishi et al. [37]
*Ccr2* knockout blocks monocyte recruitment and attenuates streptozotocin‐induced diabetic cardiomyopathy.	Animal, causal (genetic knockout)	Tan et al. [38]
Sympathetic denervation attenuates post‐MI atherosclerosis acceleration and systemic inflammation, implicating neural input in trained immunity maintenance.	Animal, causal (chemical sympathectomy, 6‐OHDA)	Dong et al. [39]
Cardiomyocyte and endothelial *Sirt6* loss‐of‐function causes cardiac hypertrophy/heart failure with preserved ejection fraction (HFpEF) in mice; SIRT6 protein concordantly reduced in failing human myocardium.	Mouse genetic causal with concordant human observational validation	Sundaresan et al. [40]; Wu et al. [41]

**Tier B** Inferred from animal models, in vitro systems, or convergent evidence.

**Table:** 

**Mechanism/observation**	**Evidence type**	**Key references**
H3K18 lactylation persists for at least 90 days after initial stimulation in trained immune cells.	In vivo human cohort (BCG vaccination) with in vitro mechanistic confirmation; immune‐lineage, cardiac extension untested	Ziogas et al. [42]
H3K4me1 persists at decommissioned regulatory elements through monocyte‐to‐macrophage differentiation (∼6 days) following brief β‐glucan stimulation.	In vitro, β‐glucan‐trained monocyte‐to‐macrophage model	Saeed et al. [16]
Nuclear ACLY drives site‐specific histone acetylation at fibrotic gene loci; its inhibition reverts the established fibrotic phenotype even at 48 h post‐activation.	In vitro, cardiac fibroblasts	Lazaropoulos et al. [18]
Myocardial NAD^+^ depletion is documented across heart failure models with functional rescue by NAD^+^ repletion, consistent with impaired sirtuin‐mediated deacetylation.	Animal time‐course (pressure overload, dilated cardiomyopathy, HFpEF) with confirmation in human failing myocardium	Lee et al. [19]; Diguet et al. [43]; Tong et al. [20]
LVAD mechanical unloading reverses only 3.2% of heart failure‐associated DNA methylation changes after hemodynamic correction.	Human pre/post observational (hemodynamic intervention)	Liao et al. [21]
Adipose tissue retains epigenetic signatures of obesity after weight loss, associated with accelerated rebound on rechallenge in mice.	Human adipose tissue, animal rechallenge	Hinte et al. [44]
Superimposed renal failure amplifies cardiac histone modifications beyond diabetes alone.	Animal model (diabetic nephropathy)	Gaikwad et al. [45]
HDAC inhibition reverses pre‐existing diastolic dysfunction by blocking covert extracellular matrix (ECM) remodeling.	Animal model (murine HFpEF)	Travers et al. [46]
HDAC6 inhibition reverses established HFpEF with additive benefit when combined with empagliflozin.	Animal model (murine HFpEF)	Ranjbarvaziri et al. [47]

**Tier C** Testable hypotheses requiring prospective validation.

**Table:** 

**Hypothesis**	**Mechanistic rationale**	**Validation strategy**
Peripheral organ chromatin modifications directly drive cardiac chromatin remodeling through sustained mediator production	Indirect support from interventional studies (SeP knockout, AST‐120) and temporal lag patterns; direct chromatin causality not demonstrated	Cardiac‐specific epigenomic profiling before and after isolated peripheral mediator manipulation in humans or large animal models
Cofactor depletion in fibroblasts and immune cells impairs eraser function in neighboring cardiomyocytes through local NAD^+^ and α‐KG gradients.	Mechanistic plausibility from cofactor biology; no spatial demonstration in vivo	Spatial metabolomics of NAD^+^ and α‐KG gradients in stressed myocardium
SGLT2 inhibitors and GLP‐1 RAs reverse established epigenetic modifications rather than only preventing new ones	Post‐treatment durability data suggestive; epigenetic mechanism not confirmed in human tissue.	Pre/post epigenetic profiling in cardiac tissue from clinical trial populations
Clonal hematopoiesis and metabolic trained immunity converge on shared enhancer landscapes in hematopoietic progenitors.	Both drive persistent myeloid inflammation through chromatin marks; convergence vs. parallel mechanisms unknown.	Parallel chromatin profiling of *TET2*‐mutant and oxLDL‐trained progenitors within the same experimental system

### 2.1 Metabolite Sensing and Transcriptional Priming

**FIGURE 1:**
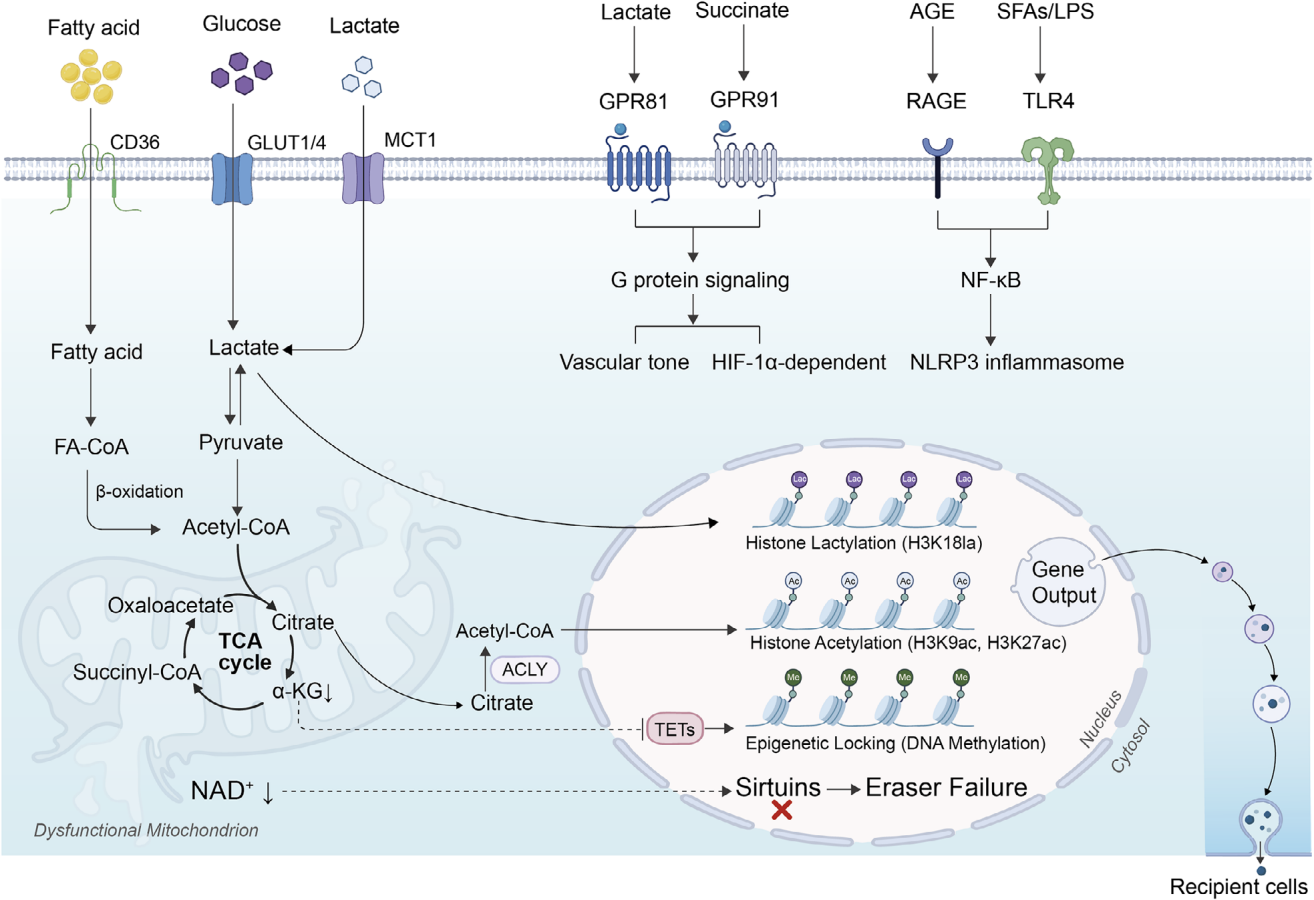
Molecular mechanisms of metabolic memory encoding and consolidation. Metabolic substrates from the circulation enter cardiac cells and feed into TCA cycle metabolism. Surface receptors sense metabolites and damage signals and drive transcriptional priming through HIF‐1α and NF‐κB. In the nucleus, metabolic intermediates serve as substrates for histone lactylation and acetylation. Persistence is reinforced by dual eraser failure: NAD^+^ depletion inactivates sirtuins, sustaining histone acetylation, while α‐ketoglutarate depletion impairs TET demethylases, sustaining DNA methylation. The encoded phenotype can spread to recipient cells through extracellular vesicles (Section 3.1). Abbreviations: ACLY, ATP citrate lyase; AGE, advanced glycation end product; α‐KG, α‐ketoglutarate; CD36, cluster of differentiation 36; FA‐CoA, fatty acyl‐coenzyme A; GLUT1/4, glucose transporter 1/4; GPR81, G‐protein‐coupled receptor 81; GPR91, G‐protein‐coupled receptor 91; H3K18la, histone H3 lysine 18 lactylation; H3K9ac, histone H3 lysine 9 acetylation; H3K27ac, histone H3 lysine 27 acetylation; HIF‐1α, hypoxia‐inducible factor 1α; LPS, lipopolysaccharide; MCT1, monocarboxylate transporter 1; NAD^+^, nicotinamide adenine dinucleotide; NF‐κB, nuclear factor κB; NLRP3, NOD‐like receptor protein 3; RAGE, receptor for advanced glycation end products; SFAs, saturated fatty acids; TCA, tricarboxylic acid; TETs, ten‐eleven translocation enzymes; TLR4, Toll‐like receptor 4. Created in BioRender.

Brief receptor engagement produces reversible signaling that normalizes when the ligand is withdrawn. Prolonged or recurrent activation, however, can establish chromatin changes that outlast the stimulus. Lactate and succinate exemplify this: G‐protein‐coupled receptor 81 (GPR81) senses extracellular lactate to modulate vascular tone and hemodynamic response [48], while G‐protein‐coupled receptor 91 (GPR91, also known as SUCNR1) senses extracellular succinate to enhance IL‐1β production in macrophages [49]; in endothelial cells, this proceeds through a GPR91/HIF‐1α/IL‐1β axis [50]. GPR91 signaling drives a hypertrophic program under ischemic conditions [51] and a fibrotic program in aging‐associated cardiac stress [52] (Figure 1). A parallel logic applies to lipid sensing: oxidized low‐density lipoprotein (oxLDL) reprograms monocytes toward trained immunity [53] discussed in Section 3.1. The temporal threshold separating reversible signaling from durable encoding has not been defined in any cardiac cell type. Receptor sensing initiates this transcriptional priming; its conversion into durable memory depends on the chromatin consolidation described next. Across these routes, sustained sensing does not merely produce a transient response but leaves the responding cells in a primed transcriptional state, one predisposed to faster or amplified reactivation on re‐exposure.

### 2.2 Chromatin Consolidation and Irreversible Modification

Durable dysfunction requires chromatin consolidation, in which reversible marks become stable configurations resistant to normalization. This operates through two complementary modes. In accumulation‐based encoding, metabolites serve as substrates for covalent histone modification. In depletion‐based encoding, cofactor exhaustion disables the erasure machinery that would otherwise remove these marks. Operationally, consolidation is the point at which metabolic normalization alone no longer reverses the marks.

#### 2.2.1 Accumulation‐Based Encoding

Histone lactylation illustrates this mode. Elevated intracellular lactate is used to deposit H3K18 lactylation, which, after myocardial infarction, activates reparative gene programs in infiltrating macrophages [13]. Lactylation produces context‐dependent outcomes across cardiac cell types: pro‐hypertrophic via H3K18la in cardiomyocytes [14], protective via non‐histone protein lactylation in fibroblasts [54], and reparative in two independent macrophage contexts [13, 55]. In cardiomyocytes specifically, a cardiomyocyte‐specific *Ldha* deletion and promoter‐level analysis show that lactate‐driven H3K18la at the *TGFB2* promoter promotes pressure‐overload hypertrophy and heart failure [56]. Its persistence after stimulus withdrawal, however, has not been characterized in any cardiac cell type, and whether the 90‐day persistence seen in trained monocytes [42] extends to cardiomyocytes or fibroblasts remains an open question.

Histone acetylation couples cardiac metabolic state to gene expression through nuclear ATP citrate lyase (ACLY), which converts citrate to acetyl‐CoA for chromatin acetylation [17]. In cardiac fibroblasts, ACLY inhibition reverses established myofibroblast differentiation even after the phenotype is fully established at 48 h [18], indicating that acetylation‐based marks at this stage remain therapeutically accessible.

In trained monocytes, a mark hierarchy governs durability. Stimulation deposits H3K27ac at inflammatory enhancers, which is lost during subsequent differentiation, whereas H3K4me1 persists and creates primed enhancers that respond faster to re‐challenge [16, 57]. This hierarchy has therapeutic consequences. Inhibitors can prevent trained‐immunity formation, and no study has yet shown they erase established H3K4me1 [58, 59]. Consistent with this, three months of statin therapy lowers lipids yet does not reverse monocyte hyperreactivity or its epigenetic marks [33]. Once marks shift from labile species such as H3K27ac to stable ones such as H3K4me1, the system resists metabolic normalization alone.

#### 2.2.2 Depletion‐Based Encoding

The durability of accumulation‐based marks depends on eraser function, which requires specific cofactors. When these cofactors are depleted, transient marks become persistent.

Sirtuins require nicotinamide adenine dinucleotide (NAD^+^) for histone deacetylation, so NAD^+^ depletion disables this eraser. In cardiac tissue, the NAD^+^/NADH ratio declines within 4 weeks of pressure overload [19], and H3K9ac accumulates as SIRT6 falls [60] (Figure 1). This is not only an experimental inference. SIRT6 protein is reduced in human failing hearts [40] and in cardiac endothelial cells from diabetic heart failure patients [41]. In HFpEF, nicotinamide phosphoribosyltransferase (NAMPT), the rate‐limiting enzyme of cardiac NAD^+^ salvage, is reduced in human biopsies, with direct NAD^+^ depletion shown experimentally [20]. The depletion is self‐reinforcing, because mitochondrial dysfunction further lowers NAD^+^. Even so, NAD^+^ repletion reverses established HFpEF in experimental models [20], indicating that this eraser failure remains therapeutically accessible.

Methylation marks are longer‐lasting and require active enzymatic reversal. Nuclear α‐ketoglutarate (α‐KG) is an essential cofactor for ten‐eleven translocation (TET) DNA demethylases [22], and its depletion during cardiac stress impairs demethylation and favors repressive chromatin (Figure 1). α‐KG supplementation can initially reverse aberrant methylation [22], but the coupling of maintenance methylation to histone deacetylation is consistent with progressively limited reversibility [61].

#### 2.2.3 The AGE Boundary

AGEs represent structural scarring rather than encoded memory. Even so, AGE–receptor for advanced glycation end products (RAGE) signaling sustains NF‐κB [62] and NOD‐like receptor protein 3 (NLRP3) [63] priming and thereby modifies chromatin accessibility. Whether this scarring persists in the heart is the relevant question here, but it has not been measured directly: AGE persistence has been quantified only in skin. DCCT/EDIC analyses show that skin collagen AGE levels were lower in the intensively treated group at DCCT closeout and predicted microvascular and subclinical cardiovascular complications for over a decade thereafter, despite glycemic convergence [31, 32]. Cardiac collagen may behave differently, since myocardial interstitial collagen turnover has been estimated at 80–120 days, substantially faster than skin collagen [64]. Cardiac AGE persistence is therefore inferred from the irreversible chemistry of Maillard crosslinks rather than measured directly.

### 2.3 Temporal Hierarchy of Memory Encoding

**FIGURE 2:**
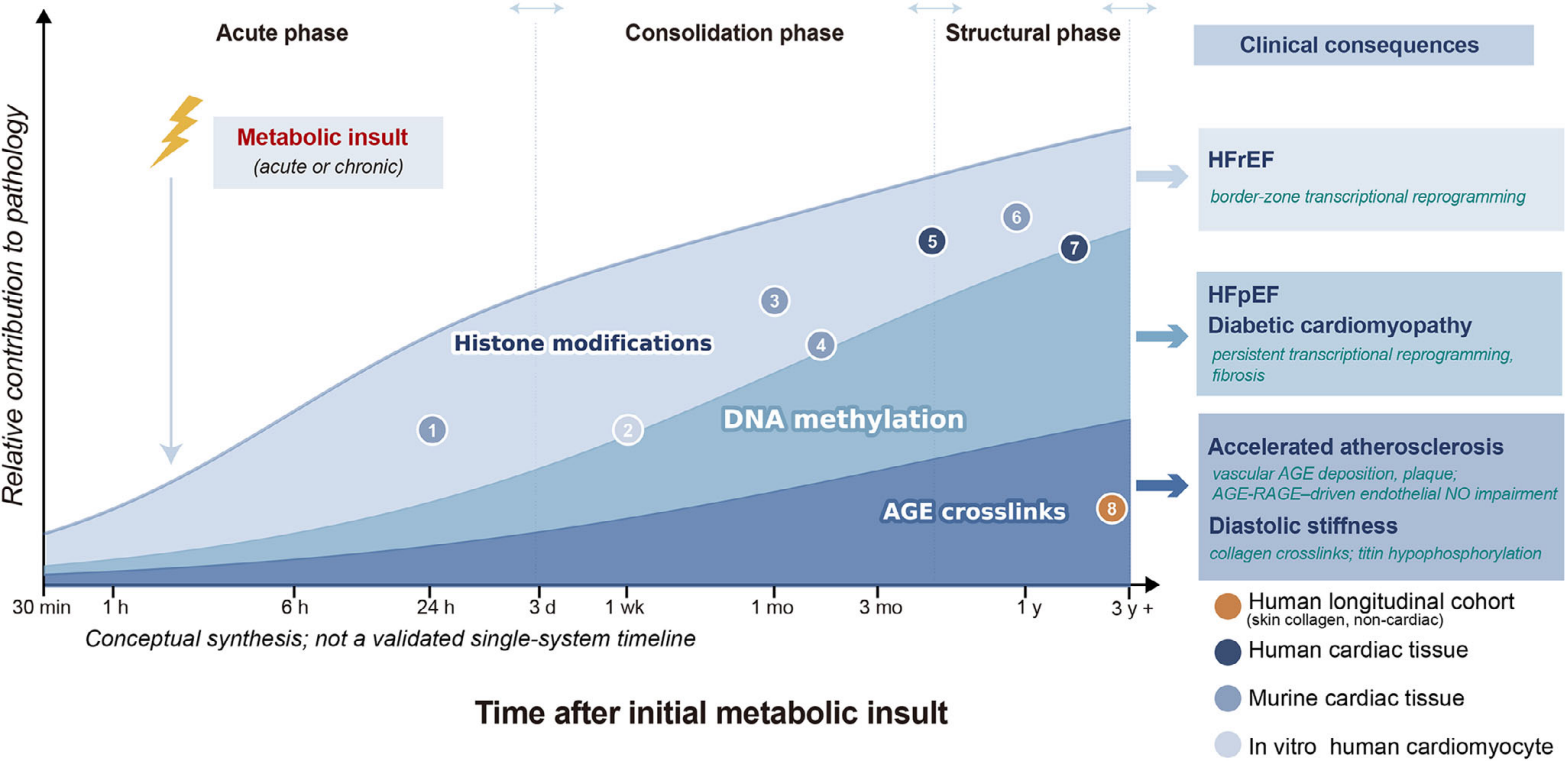
Temporal progression of cardiac metabolic memory. Schematic representation of three encoding layers that accumulate after metabolic insult: histone modifications (hours onset, conditionally reversible), DNA methylation (weeks onset, semi‐permanent), and AGE crosslinks (months onset, structural). Right panels indicate associated clinical consequences. All layers contribute to all outcomes; the grouping reflects the predominant mechanism. Phase boundaries are approximate, not discrete thresholds (Section 2.3). Numbered markers (❶–❽) denote temporal anchors from cardiac tissue, cardiomyocyte, or human cohort studies: ❶ H3K27ac super‐enhancer remodeling 24 h post‐myocardial infarction in murine cardiac tissue [65]; ❷ high‐glucose‐induced promoter demethylation in human AC16 cardiomyocytes at 7 days [66]; ❸ cardiac NAD^+^/NADH decline at 4 weeks of pressure overload in murine cardiac tissue [19]; ❹ eight‐week diabetes alters cardiac gene expression without detectable DNA methylation changes in murine cardiac tissue [67], defining the early boundary for DNA methylation accumulation; ❺ reduced *NAMPT* expression in human HFpEF myocardial biopsies [20]; ❻ persistent histone demethylation in diabetic rat hearts after intensive glycemic control [68]; ❼ persistent epigenetic factor upregulation in human and murine hearts after mechanical unloading [21, 69]; ❽ DCCT‐closeout skin AGE levels predictive of complications through EDIC year 15–16 in DCCT/EDIC participants [31, 32]. The framework is intended as an operational model for hypothesis generation rather than a fixed chronological sequence applicable to every disease context. The anchors span four evidence categories, denoted by anchor color in the figure: murine cardiac tissue (❶, ❸, ❹, ❻), an in vitro human cardiomyocyte line (❷), human cardiac tissue (❺, ❼), and a human longitudinal cohort measured in skin rather than cardiac tissue (❽). No single system spans all three phases; the unified timeline is a conceptual synthesis requiring prospective validation (Section 5.3). Abbreviations: AC16, human cardiomyocyte cell line; AGE, advanced glycation end product; HFpEF, heart failure with preserved ejection fraction; HFrEF, heart failure with reduced ejection fraction; H3K27ac, histone H3 lysine 27 acetylation; NAD^+^, nicotinamide adenine dinucleotide; NAMPT, nicotinamide phosphoribosyltransferase; NO, nitric oxide; RAGE, receptor for advanced glycation end products.

The encoding mechanisms above operate across distinct timescales, creating a temporal stratification that shapes therapeutic accessibility. Memory formation can be organized into three sequential phases defined by mark stability and reversibility (Figure 2). The three phases describe a fixed sequence of states, not fixed durations; how long each phase lasts depends on the insult. The molecular substrate that an intervention must engage changes as encoding progresses.

#### 2.3.1 Acute Phase

Rapidly reversible substrate–chromatin coupling predominates: histone acetylation and lactylation track the metabolic signal and remain vulnerable to erasure while cofactor pools are intact. Withdrawal of the stimulus at this stage permits endogenous reversal.

#### 2.3.2 Consolidation Phase

Consolidation begins when stress depletes erasers faster than they are restored. The transition is not abrupt: acetylation‐based marks can retain reversibility into early consolidation, as ACLY inhibition reverses myofibroblast differentiation even at 48 h post‐establishment [18]. For depletion‐based marks, consolidation occurs when cofactor exhaustion exceeds restoration capacity; for accumulation‐based marks, when self‐sustaining writer activity persists independently of the stimulus. The NAD^+^/NADH decline within 4 weeks of pressure overload [19] provides a quantitative anchor for onset.

#### 2.3.3 Structural Phase

This phase comprises changes for which no current intervention achieves reversal, including AGE crosslinks [70] and nephron loss; vascular calcification lacks any established clinical reversal but is increasingly characterized as an actively regulated rather than passively irreversible process [71]. Left ventricular assist device (LVAD) unloading normalizes only 3.2% of heart failure–associated DNA methylation changes [21], with persistent dysregulation of mitochondrial genes and chromatin remodelers [69]. This pattern is consistent with a consolidation or structural phase but does not distinguish them. Because unloading does not target chromatin directly, three possibilities remain open: late consolidation, sustained peripheral input, or true structural irreversibility. Resolving this will require chromatin‐directed interventions that are not yet available clinically.

#### 2.3.4 Immune Versus Cardiomyocyte Persistence

The apparent paradox of acetylation marks persisting for years reflects two different mechanisms. In immune lineages, marks in any individual monocyte are short‐lived; what persists is the bone‐marrow program that re‐establishes them on each new generation of monocytes through progenitor reprogramming [72]. Cardiomyocytes renew at under 1% per year [73], so their marks persist passively through cell longevity, although consolidation onset can be rapid, as in poly(ADP‐ribose) polymerase (PARP)‐mediated NAD^+^ depletion during heart failure [74]. The methodological consequence is direct: immune‐lineage persistence timelines cannot be used to estimate cardiomyocyte timelines, because the underlying mechanisms differ. The cardiomyocyte‐specific timescale is therefore empirically undetermined rather than long by analogy. Despite these mechanistic differences, consolidation marks the same functional threshold in both lineages: before it, withdrawing the insult permits endogenous erasure; after it, reversal requires lineage‐specific intervention.

#### 2.3.5 Falsifiable Predictions

This three‐phase hierarchy is an integrative inference and has not yet been tracked within a single cardiac system (Figure 2). Two findings would challenge it. First, serial cardiac biopsies would need to show histone acetylation, DNA methylation, and AGE accumulation emerging concurrently rather than sequentially. Second, LVAD would need to reverse a substantially higher proportion of DNA methylation changes than the 3.2% currently observed, which would place the consolidation‐to‐structural boundary later and imply a wider therapeutic window. Prospective serial cardiac epigenomic sampling is needed to confirm, revise, or refute these boundaries. For each core claim, Table S1 sets out the decisive test and the outcome that would confirm, revise, or refute it. The absence of biomarkers to stage these phases is a key translational gap.

## 3 Cellular and Systemic Determinants of Memory Persistence

**FIGURE 3:**
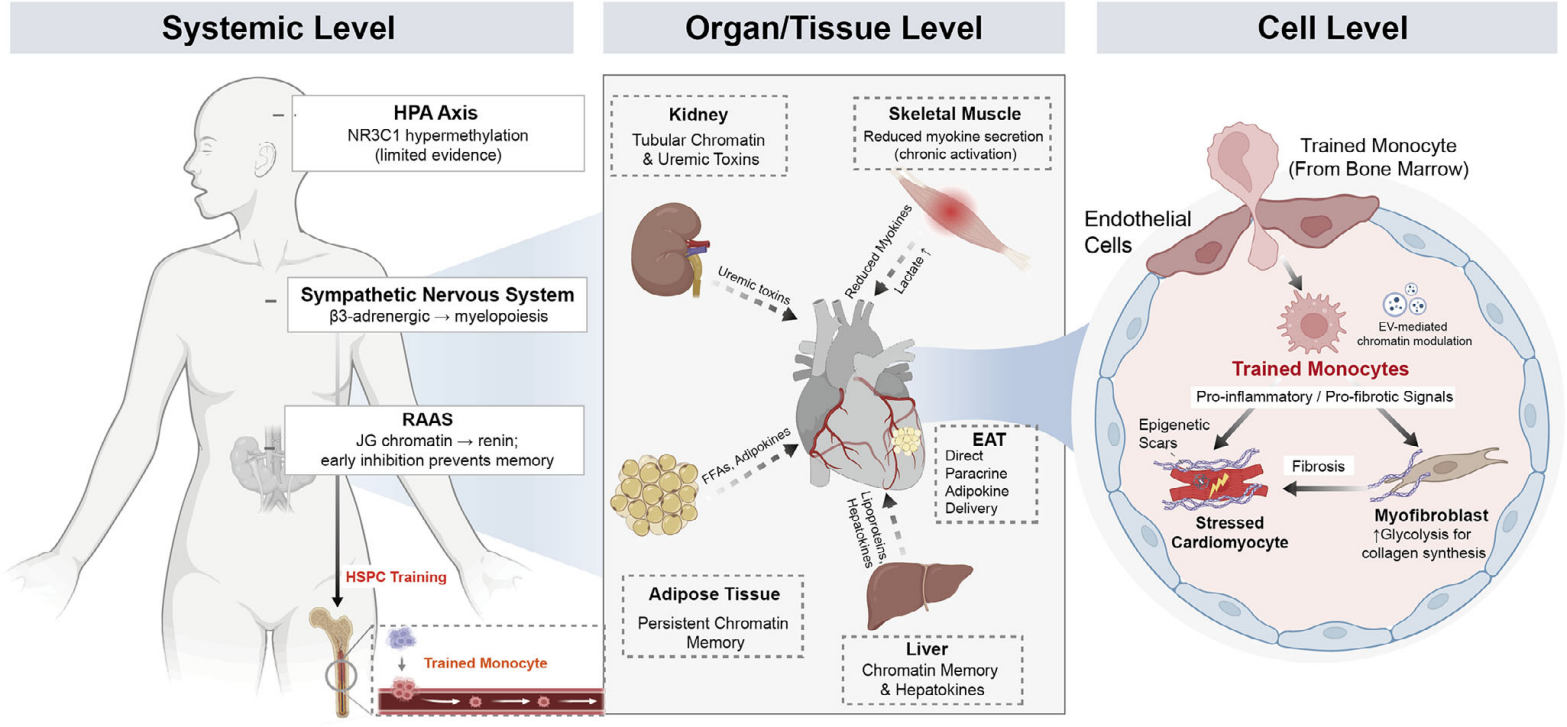
Cellular and systemic determinants of cardiovascular metabolic memory persistence. Metabolic memory is sustained across three scales (Section 3). At the systemic level (left), three neuroendocrine axes reinforce memory with differing evidence strength: the sympathetic nervous system biases bone‐marrow output toward myelopoiesis through β3‐adrenergic signaling, driving HSPC training and the release of trained monocytes; the RAAS sustains renin through juxtaglomerular chromatin modification, where early inhibition is associated with a durable repressive mark and a lasting reduction in blood pressure; and the HPA axis shows *NR3C1* promoter hypermethylation, for which evidence remains limited. At the organ/tissue level (center), peripheral organs supply persistent pathological input to the heart: the kidney (uremic toxins, tubular chromatin), adipose tissue and epicardial adipose tissue (adipokines, persistent chromatin memory), and the liver (hepatokines, chromatin memory), whereas skeletal muscle contributes through chronic activation rather than encoded memory (reduced myokines). At the cell level (right), bone‐marrow‐derived trained monocytes and EVs whose cargo can modulate chromatin‐related machinery in recipient cells contribute to pro‐inflammatory and pro‐fibrotic signaling, producing stressed cardiomyocytes and myofibroblasts with epigenetic scarring and fibrosis. Together, these scales consolidate a persistent phenotype characterized by metabolic inflexibility, mitochondrial dysfunction, epigenetic locking, and AGE crosslinks (detailed in Figures 2 and 5). Abbreviations: AGE, advanced glycation end product; EAT, epicardial adipose tissue; EV, extracellular vesicle; FFAs, free fatty acids; HPA, hypothalamic–pituitary–adrenal; HSPC, hematopoietic stem and progenitor cell; JG, juxtaglomerular; RAAS, renin–angiotensin–aldosterone system. Created in BioRender.

The molecular encoding mechanisms described in Section 2 operate within individual cells or between immediate neighbors. Cardiovascular disease, however, manifests as multi‐organ pathology. This section examines how encoded programs are sustained and reinforced across three biological scales: paracrine circuits within cardiac tissue, peripheral organ dysfunction that maintains pathological input to the heart, and systemic neuroendocrine amplification (Figure 3).

### 3.1 Cellular Level: Trained Immunity and Paracrine Metabolic Crosstalk

Within cardiac tissue, interactions among macrophages, fibroblasts, and cardiomyocytes are proposed to determine whether metabolic stress resolves or consolidates into persistent pathology. The supporting evidence derives mainly from co‐culture and animal models. When inflammatory stress persists, these cells form feed‐forward circuits: activated fibroblasts raise glycolytic flux for collagen synthesis [75], generating lactate that drives macrophage histone lactylation [13], while TGF‐β signaling and other cytokines reciprocally activate fibroblasts [76], with macrophages representing a plausible but not directly confirmed source in this context. Extracellular vesicles (EVs) add a further layer. In an in vitro model, exosomes from compound‐treated cardiac mesenchymal stem cells raised H3K27me3 in recipient HL‐1 cardiomyocytes by suppressing transcription of the demethylase UTX [77], illustrating that EV cargo can influence erasure machinery in recipient cells. Whether EVs induce chromatin changes that persist after exposure ends, however, has not been tested.

**FIGURE 4:**
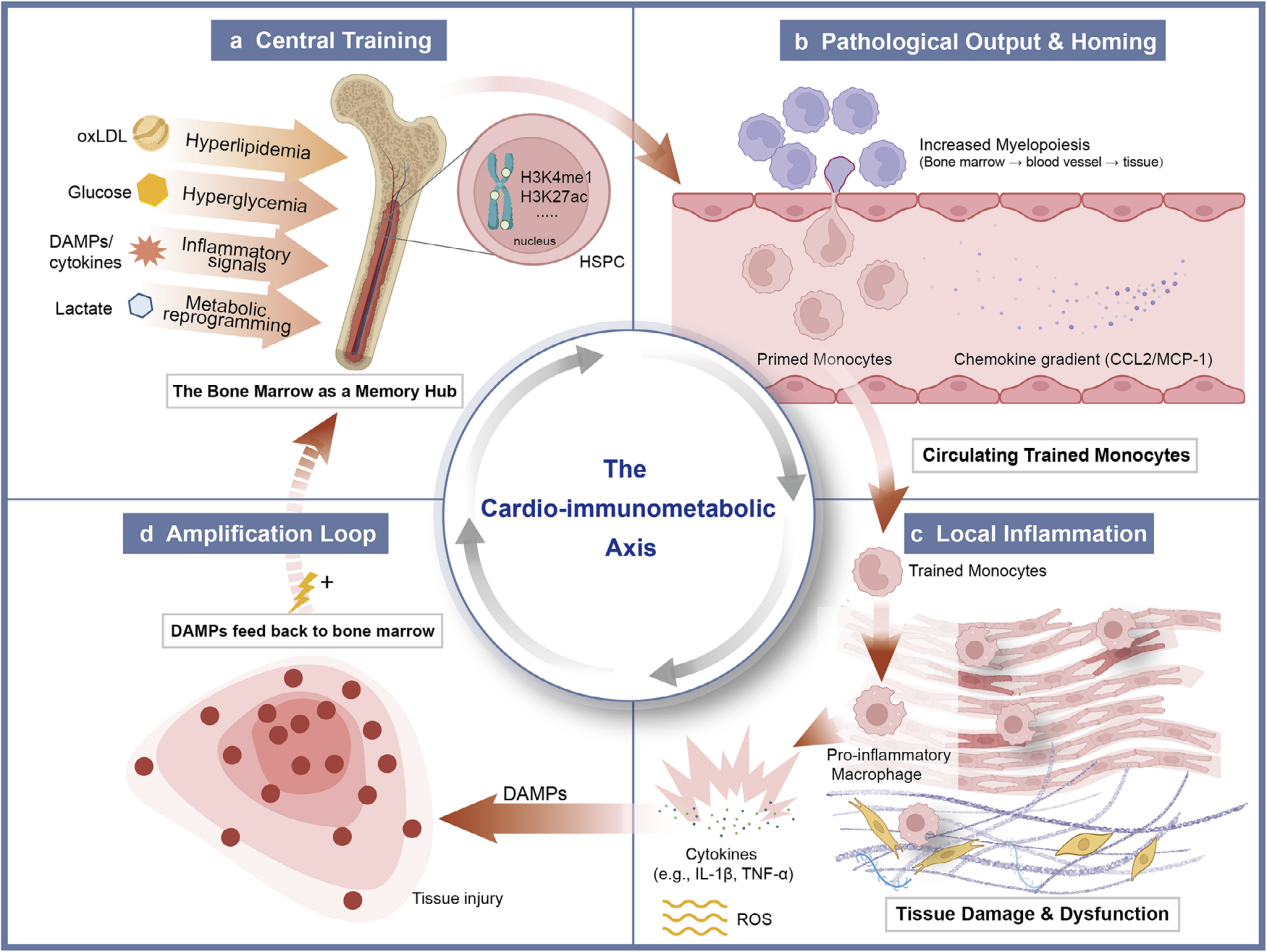
The cardio‐immunometabolic axis. A self‐reinforcing circuit through which bone marrow‐encoded immune memory perpetuates cardiac inflammation independent of ongoing metabolic stress. (a) Central training. Metabolic and inflammatory inputs (oxidized LDL, glucose, DAMPs/cytokines, lactate) reprogram hematopoietic stem and progenitor cells (HSPCs) in the bone marrow, establishing persistent H3K4me1 and activation‐associated H3K27ac at inflammatory gene loci. (b) Pathological output and homing: trained progenitors increase myelopoiesis and release primed monocytes that traffic to tissue along a CCL2/MCP‐1 chemokine gradient. (c) Local inflammation: recruited monocytes mature into pro‐inflammatory macrophages, releasing cytokines (e.g., IL‐1β, TNF‐α) and ROS that drive tissue damage. (d) Amplification loop: tissue injury releases DAMPs that feed back to the bone marrow, reinforcing the trained phenotype. Because the inflammatory source resides in the bone marrow rather than the heart, local cardiac anti‐inflammatory intervention cannot eliminate this systemic supply. Abbreviations: CCL2, C–C motif chemokine ligand 2; DAMPs, damage‐associated molecular patterns; H3K4me1, histone H3 lysine 4 monomethylation; H3K27ac, histone H3 lysine 27 acetylation; HSPCs, hematopoietic stem and progenitor cells; IL‐1β, interleukin 1β; MCP‐1, monocyte chemoattractant protein 1; oxLDL, oxidized low‐density lipoprotein; ROS, reactive oxygen species; TNF‐α, tumor necrosis factor α. Created in BioRender.

Trained immune cells continuously traffic from the bone marrow to the heart, carrying pro‐inflammatory phenotypes established through progenitor reprogramming [78, 79] (Figure 4). Because the inflammatory source resides in the bone marrow rather than the heart, local cardiac anti‐inflammatory treatment cannot eliminate this systemic supply. These cellular circuits therefore combine two components: encoded memory (trained immunity, persistent chromatin marks) and chronic activation (ongoing paracrine signaling). Distinguishing the two in vivo remains an open challenge.

### 3.2 Peripheral Organ Contributions to Cardiac Pathology

Peripheral organs acquire persistent chromatin modifications during metabolic stress and continue to expose the heart to inflammatory, lipotoxic, and uremic injury. For each axis, we indicate whether current evidence supports encoded memory, chronic activation, or a combination not yet resolved.

#### 3.2.1 The Adipose–Cardiac Axis

Adipose tissue encodes inflammatory memory that survives weight loss: adipocytes retain obesity‐associated epigenetic alterations and show accelerated regain on high‐fat re‐challenge in mice [44]. Epicardial adipose tissue delivers adipokines directly to adjacent myocardium, with no fascial barrier between them [80]. Left‐ventricular mass regresses only partially after bariatric surgery, lagging the achieved weight loss [81, 82], consistent with persistent adipose encoding, although delayed remodeling cannot be excluded. This axis thus combines encoded memory with chronic mediator input; direct demonstration that adipose mediators cause cardiac chromatin remodeling is lacking.

#### 3.2.2 The Hepatic–Cardiac Axis

The liver encodes metabolic dysfunction through stable chromatin‐accessibility changes, with epigenetic priming preceding downstream dysfunction by months [83, 84]. Selenoprotein P provides causal evidence: global knockout attenuates ischemia–reperfusion injury and hepatic re‐expression restores it, identifying liver‐derived selenoprotein P as the causal source [36], while elevated circulating levels predict adverse human heart failure outcomes [37]. Whether these hepatokines modify cardiac chromatin directly or act through intermediate signaling is unresolved.

#### 3.2.3 The Renal–Cardiac Axis

The kidney contributes through two mechanisms of divergent reversibility: tubular chromatin modifications [85, 86] and irreversible nephron loss. Superimposed renal dysfunction exacerbates cardiac histone modifications beyond diabetes alone [45], showing how organ damage lowers the threshold for cardiac consolidation. Uremic mediators are causally implicated: indoxyl sulfate activates the NLRP3 inflammasome and induces cardiac fibrosis and hypertrophy [87], and the toxin adsorbent AST‐120 improves experimental cardiac output [88]. Kidney transplantation requires roughly 2 years for left‐ventricular hypertrophy to nadir [89]. The axis spans encoded memory (tubular marks), structural scarring (nephron loss), and chronic activation (uremic toxins).

#### 3.2.4 The Muscle–Cardiac Axis

Unlike the others, skeletal muscle has not been shown to encode autonomous chromatin memory driving cardiac pathology; it acts through chronic activation, as muscle wasting reduces protective myokines such as irisin [90, 91] and musclin [92, 93]. This carries therapeutic weight: adipose‐ and liver‐directed treatment must address both encoded memory and ongoing mediator production, whereas muscle‐directed measures such as exercise and myokine therapeutics address mediator deficiency with no memory component to reverse. This axis therefore exemplifies chronic activation and should not be conflated with the encoded memory of the other axes.

#### 3.2.5 Integration

These axes operate simultaneously and amplify one another, and they decay asynchronously: hepatic chromatin changes may reverse with weight loss, adipose marks persist [44], and renal structural change approaches permanence. Single‐organ intervention therefore yields incomplete benefit, because pathological input from the other organs continues.

The remaining gap is the final causal step from periphery to heart. Persistent peripheral chromatin modifications are well documented [44, 83, 84, 85, 86], and reducing peripheral mediator burden improves cardiac outcomes [88, 94], but direct proof that peripheral mediators drive specific chromatin modifications in human cardiomyocytes is lacking. Two models remain compatible with the evidence. In a peripheral‐driven model, sustained mediator exposure directly causes cardiac chromatin remodeling, and treating the peripheral source would suffice. In a parallel‐encoding model, the heart encodes independent modifications to local stress while peripheral organs encode their own, so both must be treated. Distinguishing them requires cardiac‐specific epigenomic profiling before and after isolated peripheral mediator manipulation. Notably, prior syntheses leave this gap open as well: Chen et al. [11] review organ‐intrinsic epigenetic memory in diabetic complications but do not address the cross‐organ step from peripheral mediators to cardiac chromatin remodeling. This is the central unresolved step in the peripheral‐to‐cardiac causal chain.

### 3.3 Neuroendocrine Amplification

The sympathetic nervous system offers the strongest evidence for neuroendocrine memory. Chronic norepinephrine biases hematopoietic output toward myelopoiesis through β3‐adrenergic signaling in the bone‐marrow niche [95], and separately regulates hematopoietic stem cell egress from the bone marrow via β2‐adrenergic signaling [96]; long‐term hematopoietic stem cells acquire long‐term epigenetic memory at myeloid enhancers [97]. Bone marrow from hyperglycemia‐exposed donors accelerates atherosclerosis in normoglycemic recipients [34], and sympathetic denervation attenuates post‐infarction atherosclerosis and systemic inflammation, implicating neural input in the maintenance of trained immunity [39].

The renin‐angiotensin‐aldosterone system (RAAS) contributes to memory both systemically and locally. Juxtaglomerular chromatin modifications sustain renin production [98], and a four‐week course of RAAS inhibition in young spontaneously hypertensive rats (SHR) is associated with increased methylation at the renin locus, including its promoter region, with blood pressure remaining lower six weeks after treatment withdrawal [99], suggesting that early intervention can install a durable repressive mark at this axis. Evidence for hypothalamic‐pituitary‐adrenal (HPA) axis encoding is weaker, limited to an association, specific to patients with comorbid depression, between *NR3C1* promoter hypermethylation and post‐acute coronary syndrome (ACS) events [100].

These systems reinforce one another through transcriptional crosstalk and resist single‐target interruption. β‐blockers, ACE inhibitors, and mineralocorticoid receptor antagonists block ongoing signaling, but whether they erase the underlying chromatin marks is untested. A related β2‐adrenergic model shows that concurrent β2‐adrenergic blockade prevents the phenylacetylglutamine (PAGln)‐induced trained‐immunity inflammatory phenotype, although whether it erases the underlying marks was not tested [101]. This gap may explain why withdrawing neurohormonal blockade is associated with clinical deterioration for several agent classes [102], although the reported patterns are heterogeneous. The association nonetheless motivates indefinite therapy and points to the chromatin‐directed strategies discussed in Section 5.

## 4 Disease‐Specific Manifestations of Metabolic Memory

**FIGURE 5:**
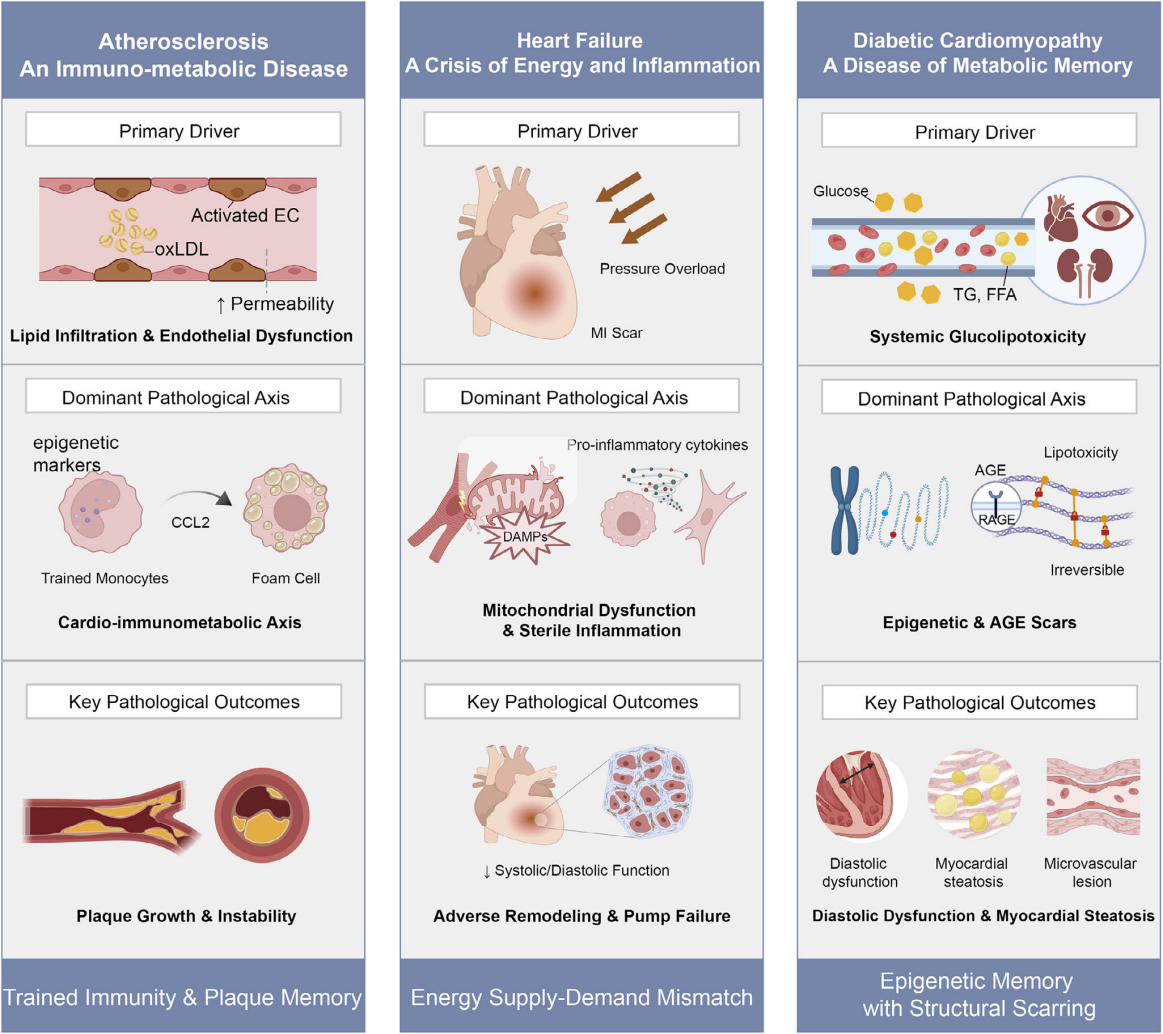
Disease‐specific manifestations of metabolic memory. Three cardiovascular diseases engage distinct pathological mechanisms while converging on shared downstream pathways. Each column shows three levels: primary driver, dominant pathological mechanism, and key outcomes (Section 4). Atherosclerosis is driven by oxidized LDL infiltration and endothelial dysfunction, with trained monocytes progressing to plaque foam cells. Heart failure reflects a convergence of cardiac injury and systemic metabolic inflammation onto a shared axis of mitochondrial dysfunction and sterile inflammation, driving adverse remodeling. Diabetic cardiomyopathy arises from glucolipotoxicity, sustained by three interlocking mechanisms independent of glycemic control: epigenetic reprogramming, AGE–RAGE signaling, and lipotoxicity. Each disease is defined by a distinct memory signature, shown at the foot of each column: trained immunity and plaque memory, an energy supply–demand mismatch, and epigenetic memory with structural scarring. Abbreviations: AGE, advanced glycation end product; CCL2, C–C motif chemokine ligand 2; DAMPs, damage‐associated molecular patterns; EC, endothelial cell; FFA, free fatty acid; MI, myocardial infarction; oxLDL, oxidized low‐density lipoprotein; RAGE, receptor for advanced glycation end products; TG, triglyceride. Created in BioRender.

The encoding and persistence mechanisms described in Sections 2 and 3 predict that cardiovascular diseases sharing metabolic origins will exhibit multiplicative rather than additive risk. Diabetes combined with myocardial infarction produces a 3.7‐fold mortality risk, nearly double the 1.9‐fold risk of diabetes alone [103]. HFpEF patients average approximately five comorbidities in large registry cohorts [104], and approximately 30% of coronary artery disease patients without a known diabetes diagnosis have undetected diabetes on systematic screening [105]. The following sections examine how these shared mechanisms manifest with disease‐specific patterns in atherosclerosis, heart failure, and diabetic cardiomyopathy (Figure 5).

### 4.1 Atherosclerosis: Trained Immunity and Plaque Macrophage Memory

Atherosclerosis exemplifies a disease in which trained immunity establishes inflammatory memory months before monocytes reach arterial plaques. Metabolic risk factors including Western diet and diabetes induce persistent chromatin marks at inflammatory gene loci in bone marrow progenitors [9], as described in Sections 2.2 and 3.1. Western diet feeding induces NLRP3‐dependent epigenomic changes in granulocyte‐monocyte progenitors that persist after dietary normalization [106]. Diabetic dyslipidemia amplifies this priming: glycated LDL is  more susceptible to oxidation [107] and its advanced glycation product engages macrophage Toll‐like receptor 4 (TLR4), RAGE, and CD36 [108], converging with trained immunity to increase atherosclerosis risk.

Once recruited to plaques, lipid‐loaded macrophages undergo metabolic reprogramming toward aerobic glycolysis, redirecting glucose‐derived carbon through ACLY to generate acetyl‐CoA that drives histone acetylation at inflammatory gene loci [17, 109, 110]. Succinate accumulation stabilizes HIF‐1α [111], and has been proposed to inhibit α‐KG‐dependent demethylases, creating metabolic inflexibility: histone deacetylase (HDAC) activity restricts fatty acid oxidation in inflammatory macrophages [112], and NF‐κB‐driven [113], HDAC‐ associated suppression of PGC‐1α (demonstrated in cardiomyocytes and plausibly extending to lipid‐loaded macrophages) further limits restoration of fatty acid oxidation capacity  [114]. This epigenetic suppression resists lipid normalization, as reflected by the persistent residual inflammatory risk observed in approximately one‐third of post‐ACS patients despite intensive lipid‐lowering therapy [115].

Impaired efferocytosis through ADAM17‐mediated MerTK cleavage [116] and tumor necrosis factor α (TNF‐α)‐induced CD47 upregulation [117] creates additional feed‐forward amplification, expanding necrotic cores through secondary necrosis. Clonal hematopoiesis of indeterminate potential (CHIP) provides a direct example: such carriers have an approximately 1.9‐fold increased coronary heart disease risk [118], and *Tet2*‐deficient cells accelerate atherosclerosis in mouse models through enhanced NLRP3‐mediated IL‐1β secretion [119].

This NLRP3‐dependent mechanism is not shared by all CHIP driver mutations. *ASXL1* mutations accelerate atherosclerosis through a non‐epigenetic pathway: wild‐type ASXL1 suppresses IRAK1–TAK1 interaction in the cytoplasm, and mutant ASXL1 loses this regulatory function, activating NF‐κB independently of chromatin remodeling [120]. *JAK2* V617F clones engage AIM2 rather than NLRP3 inflammasome signaling [121], with allele burdens as low as 1.5% sufficient to promote plaque instability [122]. These findings demonstrate that CHIP driver mutations converge on vascular inflammation through non‐overlapping molecular routes, complicating any unified chromatin‐directed therapeutic strategy. Whether clonal hematopoiesis and metabolic trained immunity converge on shared enhancer elements or activate distinct gene programs remains unresolved [121], with direct implications for whether a single chromatin‐directed intervention could address both. These distributed memory mechanisms contribute to the residual cardiovascular risk observed despite intensive lipid‐lowering therapy [123, 124], motivating the anti‐inflammatory and chromatin‐directed strategies examined in Section 5.

### 4.2 Heart Failure: Phenotype‐Specific Memory Patterns

Heart failure illustrates how metabolic memory consolidates into organ‐level dysfunction resistant to conventional hemodynamic interventions. Two distinct patterns emerge.

#### 4.2.1 HFrEF (Heart Failure With Reduced Ejection Fraction): Local Epigenetic Scars

Following myocardial infarction, border zone cardiomyocytes undergo profound transcriptional reprogramming within days, with thousands of regulatory elements switching from homeostatic to stress‐responsive pathways [125, 126]. Spatial transcriptomics reveals two distinct border zone populations, one extending hundreds of micrometers into surviving myocardium and a thinner population confined to tens of micrometers along the infarct boundary [127]. The metabolic changes in these zones are locked in through NAD^+^ depletion and sirtuin failure mechanisms (Section 2). LVAD unloading reverses only 3.2% of heart failure‐associated DNA methylation changes [21]. Mitochondrial genes comprise approximately 40% of the persistently downregulated transcripts in mice, and chromatin remodeling factors such as HDACs (e.g., *HDAC4*), *SMARCA2*, and *BRD4* remain persistently upregulated [69, 128]. This persistent chromatin remodeler expression after hemodynamic correction is among the strongest evidence that heart failure involves encoded memory.

Myocardial infarction also reprograms bone marrow. Transplanting bone marrow from heart failure mice into healthy recipients caused spontaneous cardiac dysfunction within months [35]. This creates a self‐reinforcing circuit where cardiac injury trains bone marrow progenitors, and trained monocytes continuously sustain cardiac inflammation, as described in Section 3.1 and Figure 4.

#### 4.2.2 HFpEF: Systemic Metabolic Convergence

HFpEF differs fundamentally from HFrEF, reflecting systemic metabolic disease more than discrete cardiac injury. Central adiposity exceeds 95% prevalence in HFpEF populations [129], contributing to systemic inflammation that impairs microvascular endothelial function and reduces nitric oxide bioavailability [130, 131]. A second, memory‐encoding mechanism converges here: mitochondrial dysfunction develops through SIRT3 downregulation and NAD^+^ depletion‐driven protein hyperacetylation; reduced *NAMPT* expression is shown in human HFpEF biopsies, and direct NAD^+^ reduction is measured in experimental models [20]. NAD^+^ repletion reverses established HFpEF in these models, providing proof‐of‐concept that depletion‐based encoding is therapeutically accessible during the consolidation phase. Ketone‐based intervention engages the same mitochondrial substrate from a second direction: in a composite HFpEF model, β‐hydroxybutyrate lowers the acetyl‐CoA pool and mitochondrial hyperacetylation and antagonizes NLRP3 inflammasome assembly on hyperacetylated mitochondria, attenuating IL‐1β/IL‐18 output and fibrosis [132]. This links depletion‐based mitochondrial encoding to the inflammatory amplification that sustains HFpEF.

Beyond this anti‐inflammatory action, β‐hydroxybutyrate also serves as a substrate for histone β‐hydroxybutyrylation: after myocardial infarction, H3K9bhb at the *CPT1A* locus has been reported to drive an angiogenic reparative program in cardiac endothelial cells [25]. Whether this mark persists after the metabolic stimulus resolves remains untested.

From the perspective of metabolic memory, the critical distinction is that HFpEF engages the inter‐organ network described in Section 3.2 at multiple levels simultaneously. Adipose chromatin memory sustains inflammatory gene programs after weight loss [44], hepatic chromatin modifications maintain pathological liver output [83, 84], and in murine models, superimposed renal dysfunction amplifies cardiac histone modifications beyond diabetes alone [45]. Whether these peripheral chromatin states drive corresponding modifications in human cardiomyocytes remains undemonstrated. These peripheral inputs are hypothesized to converge on a heart already compromised by intrinsic mitochondrial encoding. In advanced disease, this distinction blurs: HFrEF develops systemic metabolic derangements through bone marrow reprogramming and organ dysfunction, while HFpEF acquires focal myocardial fibrosis, creating phenotypic convergence that complicates stage‐specific intervention. This multi‐level convergence may explain why neurohormonal agents reduce mortality in HFrEF but not in HFpEF, and why agents addressing metabolic substrates achieve benefit where hemodynamic manipulation alone fails.

### 4.3 Diabetic Cardiomyopathy: Convergent Memory and Feed‐Forward Amplification

Diabetic cardiomyopathy illustrates how metabolic memory mechanisms converge to create pathology greater than the sum of individual parts. Unlike atherosclerosis where memory concentrates in plaques, or HFrEF where injury remains localized, diabetic cardiomyopathy distributes memory across the entire system through three interlocking mechanisms.

First, hyperglycemia establishes durable epigenetic reprogramming, glycemic memory in its original sense. *TXNIP* and other inflammatory genes undergo differential DNA methylation during the DCCT intervention period, notably 3′‐UTR hypomethylation, and these changes persist throughout EDIC follow‐up despite glycated hemoglobin (HbA1c) convergence [8], consistent with glucose‐independent maintenance of an inflammatory program. Second, AGEs accumulate at substantially higher levels in diabetic hearts [133], although direct human cardiac measurements are limited by tissue accessibility. Titin‐based cardiomyocyte stiffening develops through hypophosphorylation of the stiff N2B titin isoform raising passive tension [134], and AGE‐RAGE activation impairs nitric oxide bioavailability and increases oxidative stress in endothelial cells [135]. As discussed in Section 2.2, DCCT/EDIC data show that skin collagen AGE differences established by trial closeout predicted complications for over a decade despite glycemic convergence [31, 32]; in skin, this difference is likely to persist given the irreversible crosslinking chemistry of collagen AGEs [70], whereas whether cardiac AGE differences persist comparably is uncertain given faster myocardial collagen turnover [64]. Third, lipotoxicity creates metabolic inflexibility through sustained cluster of differentiation 36 (CD36) relocalization to the sarcolemma [136], ceramide‐driven mitochondrial apoptosis [137], and PKC‐ε‐mediated impairment of insulin signaling [138]. Together, these lipotoxic changes render substrate metabolism inflexible in the diabetic myocardium [139].

These three mechanisms activate feed‐forward inflammatory circuits that amplify pathology independently of glucose levels. AGE‐RAGE signaling provides the NLRP3 inflammasome priming signal [63], while dysfunctional mitochondria generate reactive oxygen species (ROS) that increase TXNIP, which in turn activates NLRP3 [140]. Multiple positive feedback loops between RAGE signaling, inflammasome activation, and inflammatory cytokine production stabilize this circuit into a self‐perpetuating state [62, 141, 142, 143]. *S100A9* and *S100A12* acquire activating histone marks at their promoters, with *S100A12* expression persisting for up to 6 days after return to normoglycemia [144]. Monocytes and macrophages accumulate in murine diabetic myocardium through CCR2‐mediated recruitment, and *Ccr2* deletion attenuates diabetic cardiomyopathy [38], consistent with a contribution of recruited, trained monocytes to disease progression.

Memory in diabetic cardiomyopathy is not confined to chromatin. Hexosamine‐flux‐driven protein O‐GlcNAcylation provides a non‐chromatin route to metabolic persistence: sustained elevation of cardiomyocyte O‐GlcNAc alone, without additional metabolic stress, is sufficient to produce cardiac hypertrophy, mitochondrial dysfunction, fibrosis, and diastolic dysfunction resembling the diabetic phenotype [145]. O‐GlcNAcylation modifies serine and threonine residues of signaling and transcriptional regulators rather than histone tails, so it lies outside the chromatin‐modification scope of Table 1. It nonetheless reinforces a broader principle: metabolic perturbation persists through more than one modification class, not chromatin marks alone.

The convergence of epigenetic consolidation, AGE accumulation, and trained immunity creates distributed memory resistant to glycemic normalization. This may explain why the active cardiovascular benefit of intensive glycemic control in DCCT/EDIC waned after about a decade, leaving only a smaller cumulative legacy [2], and why effective intervention requires multi‐target strategies addressing metabolic, inflammatory, and structural encoding simultaneously.

## 5 Therapeutic Implications: Matching Intervention to Substrate and Encoding Stage

Metabolic memory encodes transient perturbations into stable programs across distributed networks. When therapies target circulating biomarkers without addressing these encoded substrates, they normalize measurements while leaving pathology intact. Table 2 classifies interventions by this criterion. Most interventions in Table 2 engage ongoing pathological drivers rather than encoded memory marks. The table therefore assembles evidence for the substrate‐alignment principle in cardiovascular therapeutics generally; its extension to encoded memory, which no listed agent has yet shown to reverse in human myocardium, remains a working hypothesis.

**TABLE 2:** Classification of cardiovascular interventions by substrate‐causality status. Interventions are organized by the causality status of the engaged substrate, the axis on which substrate‐alignment operates; the three groups are labeled in the table. Agents whose hypothesized substrate is disproven, pleiotropic, or not effectively engaged are included because their failure delimits the principle, showing which substrates do not sustain benefit. No listed agent has demonstrated reversal of an encoded memory substrate in human myocardium, so established clinical benefit is distinct from a demonstrated memory‐reversal mechanism. Two boundary cases fall outside these categories: apabetalone engages chromatin as a mark reader rather than the writer or eraser substrate that establishes memory, and its outcome trial was null; sacubitril/valsartan engages a neurohormonal substrate outside the memory framework and is included as an external contrast.

Intervention	Substrate status	Mechanism/substrate engaged	Clinical outcome	Key interpretation
**Substrate independently validated as causal (established before the outcome trial)**				
Colchicine	Independently validated	NLRP3 inflammasome dampening; engages innate immune substrate	31% MACE (LoDoCo2) [146]; 23% post‐MI (COLCOT) [147]	NLRP3 pathway modulation may contribute to clinical benefit; findings are convergent with canakinumab [148]
Statins (high intensity)	Independently validated	HMG‐CoA reductase inhibition, LDL‐C lowering; engages LDL‐driven atherogenesis	22% MACE per 1 mmol/L LDL‐C (CTT) [149]	Benefit proportional to LDL‐C lowering [149]; substrate validated by Mendelian randomization [150]
PCSK9 inhibitors	Independently validated	LDL‐C lowering via hepatic LDL‐receptor upregulation; engages LDL‐driven atherogenesis	∼15% MACE (FOURIER/ODYSSEY) [123, 124]	Single‐target success through a validated causal pathway
Ezetimibe	Independently validated	NPC1L1 inhibition, reduced cholesterol absorption; engages LDL‐driven atherogenesis	HR 0.94 (0.89–0.99; IMPROVE‐IT) [151]	Confirms LDL as causal substrate
Bempedoic acid	Independently validated	Hepatic ACLY inhibition, LDL‐C lowering; engages LDL‐driven atherogenesis	13% MACE (HR 0.87; CLEAR Outcomes) [152]	Extends LDL causality beyond the statin mechanism (statin‐intolerant)
Canakinumab	Independently validated	Selective IL‐1β neutralization; engages IL‐1β/NLRP3 axis	15% MACE (CANTOS) [153]	*IL6R* Mendelian randomization supports causality of the downstream IL‐6 effector [154, 155]; benefit LDL‐independent [153]
**Substrate retrospectively inferred (outcome‐validated benefit; substrate not independently validated)**				
SGLT2 inhibitors	Retrospectively inferred	Ketone metabolism, natriuresis, anti‐inflammatory effects; proposed to engage metabolic/inflammatory substrate	26% HFrEF HF events [156]; 21% HFpEF [157]	Dominant substrate (metabolic, hemodynamic, or epigenetic) remains undefined [156, 157]
GLP‐1 receptor agonists	Retrospectively inferred	Weight loss, anti‐inflammatory signaling, myelopoiesis modulation; proposed to engage systemic metabolic‐inflammatory substrate	20% MACE in obesity (SELECT) [158]; 14% in diabetes [159]	Benefit exceeds weight loss [160, 161], necessary substrate not identified
Semaglutide (HFpEF)	Retrospectively inferred	As above; proposed to engage adipose‐driven inflammatory substrate	Reduced symptoms, CRP, weight (STEP‐HFpEF) [162]	Addresses symptomatic and inflammatory substrates in obesity‐related HFpEF
Tirzepatide	Retrospectively inferred	Dual GLP‐1/GIP receptor agonism; proposed to engage metabolic/adipose substrate	38% CV death/HF (HR 0.62; SUMMIT) [163]	CV death alone not significant; benefit concentrated in HF events
Finerenone	Retrospectively inferred	Nonsteroidal MRA; proposed to engage inflammatory/fibrotic substrate	Reduced HF events in HFpEF (FINEARTS‐HF) [164]	Benefit distinct from hemodynamic effects of steroidal MRAs
**Substrate disproven, pleiotropic, or not effectively engaged**				
CETP inhibitors	Disproven	Raises HDL‐C quantity; does not engage a causal atherogenic substrate	Harm (torcetrapib) [165]; neutral (evacetrapib, dalcetrapib) [166, 167]; 9% (anacetrapib) [168]	Mendelian randomization does not support HDL‐C quantity as causal [169, 170]; residual benefit attributable to LDL‐C lowering [170]
TNF‐α antagonists (HF)	Pleiotropic	Non‐selective TNF‐α suppression; engages both pathological TNFR1 and protective TNFR2	Harm in HFrEF (HR 2.84; ATTACH) [171]	Net harm reflects opposing TNFR1/TNFR2 effects [172]
Methotrexate (low‐dose)	Not engaged	Broad anti‐inflammatory; does not engage the IL‐1β/NLRP3 axis	No benefit (HR 0.96; CIRT) [173]	Did not engage the IL‐1β/NLRP3 axis; hsCRP and IL‐6 unchanged [173, 174]
**Boundary cases: substrate does not fit the causality classes**				
Apabetalone (BET inhibitor)	Chromatin (reader node)	BET bromodomain inhibition; engages chromatin as a mark reader	Primary MACE not met (BETonMACE) [175, 176]	Chromatin engaged yet outcome‐neutral; acts on a mark reader, not a writer or eraser, so does not test the writer‐directed strategy
Sacubitril/valsartan	Non‐memory substrate	Neprilysin inhibition + ARB; engages natriuretic‐peptide/neurohormonal substrate	20% CV death/HF, HFrEF (PARADIGM‐HF) [177]; not met in HFpEF (RR 0.87, 0.75–1.01; PARAGON‐HF) [178]	External contrast: benefit tracks neurohormonal‐substrate dominance (PARADIGM vs. PARAGON); does not engage encoded memory

*Note*: No agent listed has demonstrated reversal of an encoded epigenetic memory substrate in human myocardium.

**Abbreviations**: ACLY, ATP citrate lyase; ARB, angiotensin receptor blocker; BET, bromodomain and extraterminal; CETP, cholesteryl ester transfer protein; CRP, C‐reactive protein; CTT, Cholesterol Treatment Trialists; CV, cardiovascular; GIP, glucose‐dependent insulinotropic polypeptide; GLP‐1, glucagon‐like peptide‐1; HDL‐C, high‐density lipoprotein cholesterol; HF, heart failure; HFpEF/HFrEF, heart failure with preserved/reduced ejection fraction; HMG‐CoA, 3‐hydroxy‐3‐methylglutaryl coenzyme A; HR, hazard ratio; hsCRP, high‐sensitivity CRP; IL, interleukin; LDL‐C, low‐density lipoprotein cholesterol; MACE, major adverse cardiovascular events; MI, myocardial infarction; MRA, mineralocorticoid receptor antagonist; NLRP3, NOD‐like receptor protein 3; NPC1L1, Niemann‐Pick C1‐like 1; PCSK9, proprotein convertase subtilisin/kexin type 9; RR, risk ratio; SGLT2, sodium‐glucose cotransporter 2; TNF‐α, tumor necrosis factor α; TNFR, TNF receptor.

### 5.1 Classification of Interventions by Substrate Alignment

No completed randomized trial has yet used epigenetic persistence as a prespecified endpoint; the discussion below is therefore framed to guide the design and interpretation of such trials.

We propose a framework for classifying cardiovascular interventions by the alignment between their mechanism and an identified pathological substrate. The framework distinguishes two categories that differ fundamentally in evidentiary status and must not be conflated. The first, the independently validated category, is one in which the targeted substrate was established as causal before the outcome trial, through Mendelian randomization or convergent genetic and interventional evidence; here the framework has genuine prospective predictive power. The second, the retrospectively inferred category, comprises agents with demonstrated randomized controlled trial (RCT) benefit whose dominant engaged substrate is assigned only after the fact. For this category, substrate assignment generates hypotheses that require prospective mechanism‐stratified validation and should not be read as causal confirmation.

This classification makes explicit where causal knowledge in cardiometabolic therapeutics currently ends. The framework is consistent with past outcomes for both categories and generates testable predictions, but the predictions it generates for the retrospectively inferred agents have not yet been independently tested. Failure to maintain this distinction has contributed to premature claims of mechanism across the cardiovascular literature, and we apply it consistently below.

#### 5.1.1 Interventions With Independently Validated Causal Substrates (LDL and IL‐1β/NLRP3 Axes)

Independent causal evidence precedes outcome trials for two pathological substrates. For low‐density lipoprotein (LDL)‐mediated atherogenesis, Mendelian randomization establishes a direct causal relationship [150]. For the IL‐1β/NLRP3 axis, two 2012 genetic analyses of the IL‐6 receptor showed that genetically reduced IL‐6 signaling lowers coronary heart disease risk [154, 155], supporting the causality of the downstream effector five years before CANTOS tested upstream IL‐1β blockade. In both cases, the causal substrate was identified by genetic evidence predating the trial, allowing these mechanisms to serve as genuine prospective tests of the substrate‐alignment principle, though causal validation of a substrate does not by itself guarantee that engaging it will yield clinical benefit.

IL‐1β neutralization with canakinumab reduces major adverse cardiovascular events (MACE) [153], and low‐dose colchicine provides complementary evidence in both stable coronary disease and post‐myocardial infarction settings [146, 147]. Both agents converge on the IL‐1β/NLRP3 innate immune axis, which is sustained by durable H3K4me1 and activation‐associated H3K27ac marks at inflammatory gene enhancers (Sections 2.2 and 4.1). Single‐target lipid‐lowering agents converge on the same principle through the LDL axis: ezetimibe [151], proprotein convertase subtilisin/kexin type 9 (PCSK9) inhibitors [123, 124], and bempedoic acid [152] each reduce MACE proportionally to LDL reduction, consistent with the Mendelian randomization prediction. The common feature across these agents is that the substrate engaged was established as causal before the trial tested the intervention.

#### 5.1.2 Interventions With Retrospectively Inferred Substrates

Sodium‐glucose cotransporter 2 (SGLT2) inhibitors reduce heart failure hospitalization [156, 179], with consistent benefit irrespective of diabetes status [156], indicating engagement of a substrate upstream of glycemic control. Glucagon‐like peptide‐1 (GLP‐1) receptor agonists reduce cardiovascular events with approximately two‐thirds of protection occurring independently of adiposity reduction [158, 160]. In HFpEF with obesity, semaglutide improves symptoms and reduces inflammation [162, 180], and tirzepatide reduces heart failure events [163]. Finerenone reduces heart failure events in HFpEF [164]. Observational and meta‐analytic data demonstrate additive benefit when SGLT2 inhibitors and GLP‐1 receptor agonists are combined [181, 182, 183, 184].

For each of these agents, the precise causal substrate engaged remains incompletely defined. For GLP‐1 receptor agonists, proposed mechanisms include direct anti‐inflammatory effects through reduced NLRP3 inflammasome activation, restoration of cardiac metabolic flexibility, and attenuation of epicardial adipose tissue inflammation; which is necessary and sufficient has not been established. In cultured human cardiomyocytes, empagliflozin prevents the hyperglycemia‐induced demethylation of the NF‐κB and *SOD2* promoters by reducing TET2 binding [66], but whether SGLT2 inhibitors modify chromatin marks in human cardiac tissue in vivo remains undemonstrated. Substrate assignments for this category should therefore be read as generating predictions for future mechanism‐stratified trials rather than as causal attributions. Promoting them to the independently validated category requires prospective evidence, such as pre/post cardiac epigenomic profiling or trials stratifying benefit by an independently measured substrate, that is not yet available.

#### 5.1.3 Interventions Targeting Non‐Causal or Pleiotropic Substrates

Cholesteryl ester transfer protein (CETP) inhibitors achieved substantial HDL elevation, but no cardiovascular benefit was attributable to the HDL‐C rise [165, 166, 167, 168]. A Mendelian randomization analysis of genetic variants primarily associated with higher HDL‐C did not detect the reduction in myocardial infarction risk predicted by observational associations [169], providing evidence against plasma HDL‐C quantity as a causal substrate in atherogenesis. TNF‐α neutralization at the higher dose increased heart failure events through non‐selective disruption of dual TNFR1/TNFR2 signaling [171, 185]. Low‐dose methotrexate failed to reduce cardiovascular events with no significant reduction in IL‐6 or hsCRP [173], indicating that the IL‐1β/NLRP3 axis was not effectively engaged. Each failure targeted a pathway that was either non‐causal, pleiotropic with opposing effects, or disconnected from the dominant pathological substrate.

#### 5.1.4 Chromatin‐Directed Therapeutics: Rationale and Limits

The success of canakinumab and colchicine among the independently validated agents carries a further implication. Both agents intercept cytokine output from the chromatin‐sustained IL‐1β/NLRP3 axis. Pharmacological inhibition during initial training prevents both chromatin remodeling and subsequent cytokine hyperresponsiveness, whereas whether intervention after mark establishment can reverse either has not been demonstrated [58, 59]. This asymmetry suggests that chromatin‐directed therapeutics targeting the upstream epigenetic marks could, in principle, offer more durable benefit than continuous cytokine neutralization, by eliminating the source of persistent inflammatory programming rather than intercepting its output. This inference is consistent with the framework but has not yet been tested in cardiovascular populations, and the relevant chromatin endpoints have not been measured in any completed cardiovascular outcome trial.

### 5.2 Evidence Hierarchy for Emerging Intervention Strategies

The three‐phase encoding model predicts that therapeutic efficacy should depend on intervention timing relative to encoding stage. This prediction has not been tested in prospective trials, and the following classification reflects evidence maturity rather than validated phase‐specific efficacy.

The classification below organizes interventions by the strength of clinical evidence for cardiovascular benefit, while separately noting whether the mechanism plausibly engages encoded metabolic memory or operates through ongoing pathological signaling. These two dimensions can dissociate, and the dissociation is itself informative: an agent may have strong RCT support for clinical benefit while its memory‐mechanism contribution remains hypothesized.

The substrate‐causality classification of Section 5.1 and the evidence‐maturity tiers of this section are two independent axes. The first asks whether the engaged substrate was established as causal before the trial; the second asks how mature the clinical evidence for benefit is. An agent occupies a position on each axis independently, so positions that appear discordant are not contradictory. For example, canakinumab and colchicine sit with the LDL‐lowering agents on the substrate axis (independently validated) yet with the SGLT2 inhibitors, GLP‐1 receptor agonists, and finerenone on the maturity axis (Tier 1), a group whose substrate status differs: substrate validation and clinical maturity are distinct properties. Table S2 sets out all agents on both axes simultaneously.

Tier 1 comprises strategies with RCT‐validated cardiovascular benefit. The LDL‐lowering agents, SGLT2 inhibitors, GLP‐1 receptor agonists, finerenone, canakinumab, and colchicine have demonstrated cardiovascular event reduction in large‐scale randomized trials. The strength of clinical evidence in this tier should be distinguished from the strength of evidence that these agents engage encoded metabolic memory rather than ongoing pathological signaling, which remains weaker. For SGLT2 inhibitors, GLP‐1 receptor agonists, and finerenone, whether benefit derives from interruption of memory‐forming processes, modulation of ongoing signaling, or both has not been established. Canakinumab and colchicine engage the IL‐1β/NLRP3 axis sustained by trained immunity chromatin marks, but whether they modify these marks themselves or only intercept downstream output remains unresolved. No agent has yet achieved RCT‐validated cardiovascular benefit through a mechanism whose engagement of encoded epigenetic memory has been independently demonstrated in human cardiac tissue, and closing this gap defines a priority target for future trials.

Tier 2 comprises strategies with preclinical or early clinical evidence that more directly target chromatin substrates. HDAC inhibition reverses pre‐existing diastolic dysfunction in animal models [46], and HDAC6 inhibition reverses established HFpEF with additive benefit when combined with empagliflozin [47]. NAD^+^ repletion has entered early clinical testing. A 180‐patient randomized trial of intravenous NAD^+^ in ischemic cardiomyopathy demonstrated significant left ventricular ejection fraction improvement at one month, with trends toward reduced heart failure rehospitalization that did not reach significance [186]. Oral nicotinamide riboside in HFrEF patients increased whole‐blood NAD^+^ and improved peripheral blood mononuclear cell mitochondrial respiration, with *NLRP3* expression inversely correlated with NAD^+^ elevation [187]. Neither trial measured cardiac epigenetic endpoints. A trial specifically examining myocardial protein acetylation in LVAD patients receiving nicotinamide riboside is underway (NCT04528004), and event‐driven outcome trials remain pending.

The bromodomain and extraterminal (BET) inhibitor apabetalone did not meet its primary MACE endpoint in the BETonMACE trial [175, 176]. This negative outcome warrants explicit consideration because apabetalone represents the most direct chromatin‐targeted intervention to reach a cardiovascular outcome trial to date, and its failure constrains the framework proposed here. Several explanations are compatible with the framework. BD2‐selective rather than pan‐BET targeting may have engaged only a subset of enhancer‐bound complexes; the post‐ACS population may have already reached late‐consolidation or structural states; achieved exposure may have been insufficient; or BRD4 may not be the rate‐limiting reader at the relevant loci. The trial did not include cardiac chromatin endpoints, leaving these possibilities unresolved. Mechanistically, apabetalone occupies the BET bromodomain to block reading of acetylated histones rather than adding or removing the mark. It therefore intercepts the downstream consequence of an existing modification without altering the encoded state, and a null effect on established disease is consistent with this mode of action. BETonMACE represents an important null result that the framework must accommodate rather than overlook, and reinforces the priority of mechanism‐stratified trials with epigenetic endpoints.

These Tier 2 approaches target chromatin modifications that persist after metabolic normalization but before irreversible structural damage; their classification reflects mechanistic directness, not yet RCT‐validated outcome benefit.

Tier 3 comprises conceptual strategies whose application to cardiovascular memory remains to be developed. RNA interference platforms [188, 189] and gene editing approaches [190] extend therapeutic durability but address gene expression rather than established epigenetic modifications. DNA methylation inhibitors can block the downstream hypermethylation and inflammatory consequences of flow‐induced endothelial *Dnmt1* upregulation in preclinical models [191], though reversal of established hypermethylation once consolidated remains to be demonstrated.

In the near term, rational combinations of Tier 1 agents targeting complementary substrates offer the most pragmatic strategy. Longer‐term, pairing metabolic modulators with Tier 2 chromatin‐directed agents may achieve multi‐substrate targeting. Realizing this potential requires two diagnostic capabilities not yet available: identification of the predominant memory substrate in individual patients, and distinction between reversible and irreversible encoding stages.

### 5.3 Diagnostic and Translational Challenges

Three gaps limit the clinical application of encoding‐stage‐matched therapy. Bulk tissue measurements obscure cellular and metabolic heterogeneity revealed by single‐cell, spatial, and metabolic‐imaging approaches [192, 193, 194, 195, 196, 197]. Static molecular snapshots cannot establish causality or determine encoding phase, though genetically encoded biosensors and hyperpolarized ^1^ ^3^C MRI are advancing toward real‐time metabolic monitoring [198, 199]. Population‐level mechanisms inadequately predict individual responses, as computational phenotyping identifies distinct HFpEF subgroups with markedly different prognoses and treatment responses [200, 201, 202, 203, 204]. Table S3 summarizes candidate markers for staging metabolic memory, including those above, with the substrate each reflects, its evidence level, and the limitation that prevents any single marker from distinguishing reversible from irreversible encoding.

Three experiments would be particularly informative for testing this framework. First, longitudinal cardiac epigenomic profiling before and after sustained NAD^+^ repletion or SGLT2 inhibition, to test whether the consolidation phase is therapeutically reversible in human myocardium. Second, parallel chromatin profiling of *TET2*‐mutant and oxLDL‐trained hematopoietic progenitors, to determine whether clonal hematopoiesis and metabolic trained immunity converge on shared enhancer landscapes. Third, cardiac‐specific epigenomic measurements before and after isolated peripheral mediator manipulation in chronic kidney disease patients, to distinguish the peripheral‐driven and parallel‐encoding models (Section 3).

## 6 Limitations

These four processes are defined operationally by the mechanism responsible for persistence after the initiating metabolic disturbance is corrected, rather than by molecular identity or tissue location. The classification partitions persistent risk by the mechanism that maintains it and is not exhaustive: additional processes may emerge. Any such process is expected either to map onto one of these four persistence‐generating mechanisms or to justify a new mechanistic category. This makes the framework extensible, but its current support remains uneven.

First, the three‐phase temporal hierarchy is synthesized across different experimental models, cell types, and species. Phase boundaries may be continuous rather than discrete, and the therapeutic window classifications in Section 5.2 depend on a model that awaits prospective validation. The specific falsifiable predictions and required validation experiments are detailed in Sections 2.3 and 5.3.

Second, most mechanistic evidence derives from rodent models, with human cardiac data limited to patients with advanced heart failure undergoing LVAD support and to HFpEF biopsies [20, 21, 69]. Longitudinal tracking of metabolic memory in human cardiomyocytes remains unavailable. Modification kinetics established in immune cells, which turn over within days, may not apply to cardiomyocytes renewing at under 1% per year [73]. A further gap concerns the causal chain from peripheral organ chromatin modifications to cardiac chromatin remodeling, which remains incomplete at its final step; resolving it would clarify whether peripheral‐directed or cardiac‐directed intervention should take priority. This is an evidentiary gap in the framework's causal chain, whereas the temporal‐hierarchy limitation above concerns the staging model's internal structure; the two are different in kind, and we do not rank one above the other.

Third, this framework draws heavily on diabetes and hyperglycemia as the paradigmatic metabolic insult. Whether non‐diabetic cardiovascular diseases engage the same temporal hierarchy with comparable therapeutic windows has not been systematically tested, and encoding kinetics may differ across metabolic insults.

Taken together, these limitations indicate that the framework should be evaluated as a structured working hypothesis, not an established theory: no trial has yet tested an epigenetic memory substrate with persistence as an endpoint, so its therapeutic implications remain to be tested directly. Its value lies in organizing heterogeneous evidence into a coherent scheme that can be systematically validated, revised, or refuted by the experiments proposed in Section 5.3.

## 7 Conclusion

The most directly demonstrated finding reviewed here is the persistence of metabolite‐driven epigenetic marks in immune cells and their resistance to metabolic normalization. Importantly, the framework's core proposal does not depend on extending this finding to the heart: persistent cardiovascular risk is separated into four mechanistically distinct processes, of which only encoded epigenetic memory is proposed to be reversible by chromatin‐directed intervention. On this basis, the temporal staging model may explain why early intervention succeeds where late intervention fails. In parallel, the substrate‐alignment principle is consistent with clinical efficacy tracking engagement of the substrate that maintains pathology, rather than normalization of a surrogate biomarker. Extending this logic from immune cells to cardiomyocytes remains to be demonstrated. If validated, the framework would shift the basis for evaluating cardiovascular intervention from biomarker normalization toward substrate engagement, and would favor matching intervention to encoding stage. Prospective studies designed to evaluate these predictions would help identify which components merit clinical translation. The value of this framework therefore lies less in proposing a universal mechanism than in providing a common language for organizing heterogeneous forms of persistent cardiovascular risk into experimentally testable mechanistic categories.

## Author Contributions

All the authors contributed to the discussion of content and reviewed and edited the manuscript before submission.

## Conflicts of Interest

The authors declare no conflicts of interest.

## Supporting information




**Supporting File**: advs77260‐sup‐0001‐SuppMat.docx.

## Data Availability

Data sharing not applicable to this article as no datasets were generated or analysed during the current study.
