# Supplementary material for: Metabolic Memory in Cardiovascular Disease: Encoding, Propagation, and Therapeutic Targeting
Source: Adv Sci (Weinh). 2026 Aug 27;13(54):e77260. doi: 10.1002/advs.77260 (PMC13521202; doi:10.1002/advs.77260)
Supplement: Supplementary file 1 [file ADVS-13-e77260-s001.docx]

# **Supplementary Information**

| **Core claim** | **Tier (see Box 1)** | **Decisive test (study and measurement)** | **Outcome that would confirm, revise, or refute** |
| --- | --- | --- | --- |
| **Three-phase temporal hierarchy.** Encoding proceeds sequentially through acute (reversible acylation), consolidation (DNA methylation), and structural (AGE crosslinking, fibrosis) phases, each defining a distinct therapeutic window. | C | Serial cardiac epigenomic and structural profiling across disease stages in a single system (human serial biopsy or longitudinal large-animal model): histone acylation, DNA methylation, and AGE accumulation at matched timepoints. | **Confirm:** marks emerge in the predicted sequence. **Revise:** phase boundaries fall at different times than modeled.  **Refute:** acylation, methylation, and AGE accumulation emerge concurrently rather than sequentially. |
| **Peripheral-to-cardiac causal coupling.** Exposure to a peripheral-organ mediator directly induces specific chromatin modifications in human cardiomyocytes (peripheral-driven rather than parallel encoding). | C | Cardiac-specific epigenomic profiling before and after isolated manipulation of a single peripheral mediator, with peripheral and cardiac compartments separated, in humans or large-animal models. | **Confirm:** defined cardiomyocyte chromatin changes follow isolated peripheral-mediator exposure. **Refute:** no cardiac chromatin change follows, indicating parallel rather than peripheral-driven encoding. |
| **Reversibility of encoded memory.** Within the acute and consolidation window, chromatin-directed intervention reverses an encoded mark and its downstream program in cardiac cells. | C | Writer- or eraser-directed intervention (for example, Set7 inhibition) in a cardiac system bearing persistent marks, applied within and beyond the predicted window: measure mark, transcriptional output, and phenotype. | **Confirm:** mark and program reverse when treated early but not late.  **Revise:** reversal remains possible later than predicted.  **Refute:** no reversal at any timepoint despite mark engagement. |
| **Irreversibility of structural damage.** Once encoding reaches the structural phase (replacement fibrosis, advanced AGE crosslinking), the substrate is no longer chromatin-reversible. | B | Paired pre/post-intervention cardiac MRI (LGE, native T1, ECV) and epigenomic sampling, comparing reversal of diffuse versus replacement fibrosis under maximal therapy. | **Confirm:** replacement fibrosis (LGE) does not regress while diffuse fibrosis (T1/ECV) can.  **Refute:** replacement fibrosis regresses, indicating a wider reversible window than modeled. |
| **Cofactor-gradient cross-talk.** Local NAD⁺ and α-ketoglutarate depletion by neighboring fibroblasts and immune cells impairs eraser function in adjacent cardiomyocytes. | C | Spatial metabolomics of NAD⁺ and α-ketoglutarate gradients in stressed myocardium, co-registered with eraser activity and mark density at single-cell resolution. | **Confirm:** eraser-impaired, mark-dense cardiomyocytes co-localize with cofactor-depleted zones.  **Refute:** no spatial relationship between cofactor gradients and mark density. |
| **Bone-marrow-sustained immune memory.** Cardiac inflammatory memory is continuously regenerated from reprogrammed bone-marrow progenitors, so local cardiac anti-inflammatory therapy cannot eliminate it. | B | Progenitor-targeted versus cardiac-local anti-inflammatory intervention, comparing durability of the cardiac inflammatory phenotype after withdrawal of therapy. | **Confirm:** only progenitor-targeted intervention produces durable resolution.  **Refute:** cardiac-local intervention alone produces durable resolution. |
| **Substrate-alignment principle (Table 2).** Clinical efficacy tracks with engagement of the causal substrate maintaining pathology, not with normalization of a surrogate biomarker. | B | Mechanism-stratified trial or pre-specified post hoc analysis testing whether benefit follows engagement of the independently validated substrate rather than surrogate normalization. | **Confirm:** benefit segregates with substrate engagement.  **Refute:** benefit is independent of the substrate engaged, tracking only the surrogate. |

**Supplementary Table S1. Per-claim validation roadmap.**

Each core claim of the framework is paired with the decisive study that would test it and the outcome that would confirm, revise, or refute it. Evidence tiers follow the Box 1 definitions. Where a claim extends an observation graded higher in Box 1 (for example, the reversal and bone-marrow-memory claims), the tier here reflects the full claim, including its therapeutic or temporal extension, which is less directly tested than the underlying observation. The revise outcome is listed only for claims with a quantitative boundary that could shift (phase timing, the reversible window); claims with a binary outcome list confirm and refute alone. The entries consolidate the falsifiable predictions in Section 2.3, the Tier C hypotheses in Box 1, and the experiments outlined in Sections 3.2 and 5.3. None of the decisive tests listed has been completed; the table is a roadmap, not a record of results.

**Abbreviations:** AGE, advanced glycation end product; α-KG, α-ketoglutarate; ECV, extracellular volume; LGE, late gadolinium enhancement; MRI, magnetic resonance imaging; NAD⁺, nicotinamide adenine dinucleotide; Set7, SET domain containing lysine methyltransferase 7.

| **Intervention** | **Substrate-causality axis**  **(Section 5.1; Table 2)** | **Evidence-maturity axis (Section 5.2)** |
| --- | --- | --- |
| Statins, ezetimibe, PCSK9 inhibitors, bempedoic acid | Independently validated (LDL axis) | Tier 1 (RCT-validated) |
| Canakinumab; colchicine | Independently validated (IL-1β/NLRP3 axis) | Tier 1 (RCT-validated) |
| SGLT2 inhibitors; GLP-1 receptor agonists; finerenone; semaglutide; tirzepatide | Retrospectively inferred | Tier 1 (RCT-validated) |
| HDAC / HDAC6 inhibition; NAD⁺ repletion | Chromatin substrate; causality not independently established | Tier 2 (preclinical/early clinical) |
| RNA interference; gene editing; DNA methylation inhibitors | Conceptual; cardiac engagement not demonstrated | Tier 3 (conceptual) |
| CETP inhibitors; TNF-α antagonists; low-dose methotrexate | Non-causal or pleiotropic | Outcome-neutral/harmful |
| Apabetalone (BETonMACE) | Boundary case (chromatin reader, not writer/eraser) | RCT, endpoint not met |
| Sacubitril/valsartan | Boundary case (neurohormonal; outside the framework) | External contrast |

**Supplementary Table S2. Agent-level crosswalk of the two classification axes.**

Each intervention occupies one position on the substrate-causality axis (Section 5.1, Table 2: whether the engaged substrate was established as causal before the outcome trial) and, independently, one position on the evidence-maturity axis (Section 5.2). The two axes are distinct properties, so positions that appear discordant across them are not contradictory: canakinumab and colchicine share the substrate axis (independently validated) with the LDL-lowering agents, yet share the maturity axis (Tier 1) with the SGLT2 inhibitors, GLP-1 receptor agonists, and finerenone, whose substrate status differs. Two boundary cases fall outside the three substrate classes: apabetalone (BETonMACE) engaged the chromatin substrate as a mark reader rather than a writer or eraser and returned a null outcome; sacubitril/valsartan engages a neurohormonal substrate outside the memory framework and is included only as an external contrast (PARADIGM-HF vs PARAGON-HF). No listed agent has demonstrated reversal of an encoded epigenetic memory substrate in human myocardium, so established clinical benefit is distinct from a demonstrated memory-reversal mechanism.

**Abbreviations:** CETP, cholesteryl ester transfer protein; GLP-1, glucagon-like peptide-1; HDAC, histone deacetylase; IL, interleukin; LDL, low-density lipoprotein; NAD⁺, nicotinamide adenine dinucleotide; NLRP3, NOD-like receptor protein 3; PCSK9, proprotein convertase subtilisin/kexin type 9; RCT, randomized controlled trial; SGLT2, sodium-glucose cotransporter 2; TNF-α, tumor necrosis factor alpha.

| **Marker or modality** | **Stage or substrate reflected** | **What it distinguishes**  **(reversible vs irreversible)** | **Current evidence level** | **Feasibility** | **Key limitations** |
| --- | --- | --- | --- | --- | --- |
| Inflammatory markers (hsCRP, IL-6) | Active chronic inflammatory input and trained-immunity output | Reflects an ongoing, modifiable input rather than fixed structural change: an engageable substrate | **Clinically established (outcome-validated):** risk and on-treatment readouts in anti-inflammatory cardiovascular trials^1^ | Routine blood assays | Non-specific to cardiac memory; reflects systemic inflammation |
| Hyperpolarized ¹³C and metabolic cardiac imaging | In vivo metabolic reprogramming (substrate flux) | Reports an active, potentially reversible metabolic state preceding structural fixation | **Human feasibility:** metabolic phenotypes shown in healthy and diabetic hearts^2,3^ | Specialized hardware; hyperpolarizer required | Limited availability; metabolic state is an indirect proxy for the encoded mark |
| Single-cell and spatial multi-omics (tissue) | Cell-resolved chromatin and transcriptional memory state within myocardium | Can in principle separate cells in a reversible mark-bearing state from those committed to fibrotic or dead fates | **Human feasibility:** cardiac atlases^4^ and border-zone mapping^5^; not yet a clinical assay | Tissue biopsy; research-grade | Invasive; not standardized or prospectively validated for staging |
| Cardiac MRI tissue characterization (native T1, ECV, LGE) | Diffuse versus replacement myocardial fibrosis | Directly separates the two ends: native T1/ECV detect diffuse, partially reversible fibrosis; LGE marks irreversible replacement scar | **Clinically established (outcome-validated):** prognostic native T1, ECV, and LGE markers^6^ | Widely available | Detects the structural endpoint, not the upstream chromatin substrate |
| Circulating cardiomyocyte-specific cfDNA methylation (e.g. FAM101A) | Cardiomyocyte death (**irreversible endpoint, not a staging marker**); tissue-of-origin and methylation state of dying cells | Marks an irreversible endpoint (cardiomyocyte death), distinct from the reversible injury that troponin cannot resolve | **Human proof-of-concept:** STEMI, sepsis^7^, and heart failure^8^ | Blood draw; established methylation panels | Reports cell death, not reversible mark state; low signal in chronic stable disease |

**Supplementary Table S3. Biomarker and staging roadmap.**

The markers below span several signal types, from circulating and imaging readouts of active, potentially modifiable disease (inflammatory markers, hyperpolarized ¹³C imaging) through tissue-level resolution of cell state (single-cell and spatial multi-omics) to established structural and cell-death endpoints (cardiac MRI, cfDNA). The central translational requirement, distinguishing reversible encoded memory from irreversible structural damage, is given its own column. No single marker yet achieves this discrimination in an individual patient; developing one is an open problem the framework defines. Markers that report an irreversible endpoint rather than a stage (cfDNA) are included to delimit the boundary the staging problem must respect. Each entry's current evidence level is labeled by maturity (human feasibility, human proof-of-concept, or clinically established with outcome validation); these labels describe each marker independently and do not run in order down the table. These modalities extend the diagnostic and translational gaps identified in Section 5.3.

**Abbreviations:** cfDNA, circulating cell-free DNA; ECV, extracellular volume; hsCRP, high-sensitivity C-reactive protein; IL-6, interleukin-6; LGE, late gadolinium enhancement; MRI, magnetic resonance imaging; STEMI, ST-elevation myocardial infarction; T1, longitudinal relaxation time.

# **References**

1	Ridker, P. M. *et al.* Antiinflammatory Therapy with Canakinumab for Atherosclerotic Disease. *N Engl J Med* **377**, 1119–1131 (2017). <https://doi.org/10.1056/NEJMoa1707914>

2	Cunningham, C. H. *et al.* Hyperpolarized 13C Metabolic MRI of the Human Heart: Initial Experience. *Circ Res* **119**, 1177–1182 (2016). <https://doi.org/10.1161/CIRCRESAHA.116.309769>

3	Rider, O. J. *et al.* Noninvasive In Vivo Assessment of Cardiac Metabolism in the Healthy and Diabetic Human Heart Using Hyperpolarized 13C MRI. *Circ Res* **126**, 725–736 (2020). <https://doi.org/10.1161/CIRCRESAHA.119.316260>

4	Litviňuková, M. *et al.* Cells of the adult human heart. *Nature* **588**, 466–472 (2020). <https://doi.org/10.1038/s41586-020-2797-4>

5	Kuppe, C. *et al.* Spatial multi-omic map of human myocardial infarction. *Nature* **608**, 766–777 (2022). <https://doi.org/10.1038/s41586-022-05060-x>

6	Zhang, H. *et al.* Prognostic Value of Myocardial T1 Mapping and Extracellular Volume Fraction in Heart Failure: A Meta-Analysis. *JACC Cardiovasc Imaging* **18**, 1203–1217 (2025). <https://doi.org/10.1016/j.jcmg.2025.06.017>

7	Zemmour, H. *et al.* Non-invasive detection of human cardiomyocyte death using methylation patterns of circulating DNA. *Nat Commun* **9**, 1443 (2018). <https://doi.org/10.1038/s41467-018-03961-y>

8	Yokokawa, T. *et al.* Clinical Significance of Circulating Cardiomyocyte-Specific Cell-Free DNA in Patients With Heart Failure: A Proof-of-Concept Study. *Can J Cardiol* **36**, 931–935 (2019). <https://doi.org/10.1016/j.cjca.2019.10.016>
